# The double-edged sword of inducible defences: costs and benefits of maladaptive switching from the individual to the community level

**DOI:** 10.1038/s41598-022-13895-7

**Published:** 2022-06-20

**Authors:** Nadja J. Kath, Ursula Gaedke, Ellen van Velzen

**Affiliations:** Department of Ecology and Ecosystem Modelling, Institute of Biochemistry and Biology, University of Potsdam, Maulbeerallee 2, 14469 Potsdam, Germany

**Keywords:** Food webs, Ecological modelling

## Abstract

Phenotypic plasticity can increase individual fitness when environmental conditions change over time. Inducible defences are a striking example, allowing species to react to fluctuating predation pressure by only expressing their costly defended phenotype under high predation risk. Previous theoretical investigations have focused on how this affects predator–prey dynamics, but the impact on competitive outcomes and broader community dynamics has received less attention. Here we use a small food web model, consisting of two competing plastic autotrophic species exploited by a shared consumer, to study how the speed of inducible defences across three trade-off constellations affects autotroph coexistence, biomasses across trophic levels, and temporal variability. Contrary to the intuitive idea that faster adaptation increases autotroph fitness, we found that higher switching rates reduced individual fitness as it consistently provoked more maladaptive switching towards undefended phenotypes under high predation pressure. This had an unexpected positive impact on the consumer, increasing consumer biomass and lowering total autotroph biomass. Additionally, maladaptive switching strongly reduced autotroph coexistence through an emerging source-sink dynamic between defended and undefended phenotypes. The striking impact of maladaptive switching on species and food web dynamics indicates that this mechanism may be of more critical importance than previously recognized.

## Introduction

Under variable environments, species with fixed trait values cannot always be well-adapted, since traits that are adaptive in certain environmental conditions are likely maladaptive in other conditions. Many species can overcome this problem by phenotypic plasticity, which allows them to adapt their trait values by behavioural, morphological or biochemical changes to different environmental conditions. This optimization of their trait values can increase the species’ fitness^1,2^ and stabilize their population dynamics which decreases their risk of extinction^3–5^.

Inducible defences are a striking example of phenotypic plasticity. They allow species to react to a changing predation pressure by only expressing the defended phenotype when predation pressure is high^6,7^. Inducible defences can be behavioural, e.g. vertical migration in zooplankton^8,9^, morphological, e.g. algae growing spines or tadpoles enlarging their body size^10,11^, or biochemical mechanisms, e.g. toxin production^12^. As defence mechanisms incur costs depending on the extent and type of the defence^13–15^, inducible defences economize these defence costs by allowing individuals to express the phenotype that is most suited to the current environmental conditions (undefended and fast-growing when predators are scarce, and well-defended when predators are abundant), thereby increasing the species’ fitness^7^.

**Figure 1:**
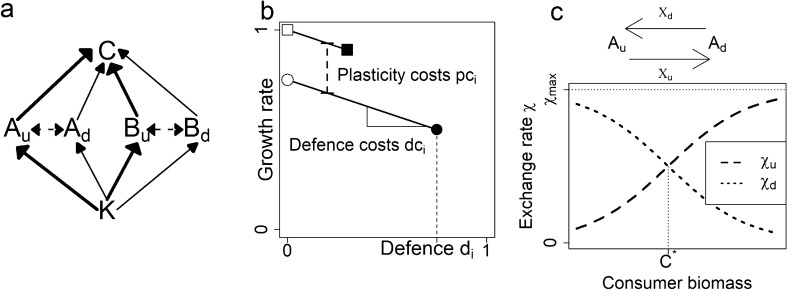
Food web structure, trade-offs, and exchange rates. (**a**) Food web of two plastic, autotrophic species *A* and *B* sharing a carrying capacity *K* and a consumer *C*. Both autotrophic species have an undefended, u, and a defended, d, phenotype. Solid arrows represent biomass fluxes and their widths indicate their relative importances. Dotted arrows indicate plastic exchange between phenotypes. (**b**) Trait space of defence and growth rate, showing how trade-offs are shaped by three key properties: level of defence *d*_*i*_, plasticity costs *pc*_*i*_ and defence costs *dc*_*i*_. White symbols denote the undefended phenotypes of a hypothetical species, black symbols the defended phenotypes of the same species. Solid lines link phenotypes of the same species. (**c**) Exchange rate χ between both phenotypes and its dependence on the consumer biomass (see Eqs. 9, 10 in “Methods”).

However, there may be costs associated with plasticity, which can arise in different ways. Plasticity costs are a reduction in a fitness-related trait, e.g. growth rate, for both the undefended and the defended phenotype of a species due to a high plasticity in expressing a range of defence, e.g. genetic information for toxicity that has to be carried along whether the phenotype produces the toxins or not^16^ (Fig. 1b). But while it makes sense to assume such costs, they were rarely found even in studies that specifically looked for them^17^. A less obvious type of cost is the possibility of maladaptive phenotype expression or maladaptive switching. Species with inducible defences need the information of the surrounding predation risk or type of predator from their environment to judge whether and which defence they need to express^18,19^. Different cues such as kairomones can be used for this decision^20^. Maladaptive switching is the risk of individuals switching from a higher-fitness phenotype to a lower-fitness one. This can happen if the cues are misinterpreted, e.g. if they are hampered by environmental influences such as CO_2_^21^, by lag times between the recognition of cues and the realization of the defence^22^, or through simple stochasticity. This may mean that individuals fail to accurately estimate current predator density, and thus make the wrong choice on whether they should display defence. Although the impact of cue reliability, and the consequent risk of maladaptive phenotype expression, for the fitness of plastic species has received extensive theoretical attention^23–26^, potential consequences of maladaptive switching for the dynamics of interacting populations have been typically neglected in investigations of inducible defences. To address this, we aim to investigate under which circumstances maladaptive switching arises, and what its consequences are for the species itself and for the other species in the food web.

A critical component of inducible defences is the speed of adaptation, which can vary depending on their defence mechanism. Behavioural strategies can be very fast, while morphological defences are rather slow, and chemical defences are somewhere in between. Differences in speed of adaptation of the prey can make the difference between the predator going extinct or not, and between stasis or oscillations^2^. A higher speed of adaptation is commonly seen as positive for the plastic organism, as either the defence is reached fast or the costs of the defence can be reduced quickly; but this depends on the assumption that switching is always adaptive, i.e. that low-fitness phenotypes switch to high-fitness phenotypes. If this assumption does not hold, faster switching may indeed be detrimental to plastic organisms implying that individuals switch more often from high to low fitness. This indicates the importance of studying whether, and under what conditions, maladaptive switching occurs.

The possibility of maladaptive switching may have consequences beyond directly lowering the fitness of the plastic species: it may impact the growth of their consumers, or lower their competitive ability compared to other prey species. Most studies take only one plastic species into account^12,21^ and therefore lack the possibility to investigate the effect of switching speed on competition between two plastic species. Here we investigate the effects of phenotypic plasticity from a community perspective, considering a small food web of two plastic autotroph species *A* and *B* having each a defended and an undefended phenotype with a joint consumer *C* and a shared carrying capacity *K* (Fig. 1a). As species cannot optimize all their traits, i.e. defence and growth rate, simultaneously, both autotrophs face a growth-defence trade-off (Fig. 1b, see “Methods” for details). Plasticity is modelled as a switching function^27^ connecting both phenotypes of a species with an exchange rate *χ*_*j*_, which depends on the consumer biomass to represent grazing pressure (Fig. 1c, see “Methods” for details). While individuals will mostly express their defended phenotype when consumer biomass is high, and their undefended phenotype when consumer biomass is low, there is a risk of maladaptive switching^27^. We indeed found a substantial amount of maladaptive switching towards undefended phenotypes, resulting in a source-sink dynamic between phenotypes, which reduced autotroph coexistence. These patterns were exacerbated by higher switching rates, which consistently provoked more maladaptive switching. Thus, counterintuitively, a higher speed of adaptation typically reduced individual fitness, lowering total autotroph biomass and increasing consumer biomass.

## Results

In our simulations for the autotrophs, we varied two of the three trade-off properties (level of defence, plasticity costs and defence costs; see Fig. 1b) at a time and kept the third one constant. This results in three constellations reflecting three different trade-offs between these properties (Table 1):

- *parallel*: trade-off between defence and plasticity costs;
- *crossing*: trade-off between defence costs and plasticity costs;
- *angle*: trade-off between defence and defence costs.

**Table 1:** Description of the three constellations *parallel*, *crossing*, and *angle* defining the position of the four phenotypes in the trait space of defence and growth rate.

	Parallel	Crossing	Angle
Property kept constant	Defence costs (slope of trade-off): 0.3	Defence levels: 0 for A_u_ / B_u_ and 0.9 for A_d_ / B_d_	Plasticity costs (overall growth reduction): 0
Non-plastic, i.e. no plasticity but four single phenotypes (“0”)			
Exchange rate *χ*_*max*_ between 10^–4^ and 10^1^ enabling plasticity			
Rigid: one phenotype per species, with mean trait values of its two phenotypes (“*”)			

Autotrophs are denoted by symbols (open triangle undefended phenotype of species A, *A*_*u*_, filled triangle defended phenotype of species A, *A*_*d*_, open circle undefended phenotype of species B, *B*_*u*_, filled circle defended phenotype of species B, *B*_*d*_, shaded triangle rigid *A*, shaded circle rigid *B*), the arrows describe the properties being varied in the simulations and the lines connect both phenotypes of a species, whereby only solid lines express actual switching between phenotypes. “0” and “*” denote the non-plastic and the rigid scenario of the respective constellation, i.e. the non-plastic scenarios are referred to as e.g. *parallel 0* in the text, the rigid ones as e.g. parallel *. The plastic scenarios are referred to by their maximum switching rate, e.g. *parallel 0.01*.

In all three constellations, the autotrophic species *B* spanned the entire defence range, i.e. it had a completely undefended phenotype *B*_*u*_ and a maximally defended phenotype *B*_*d*_. *A* either had a more limited defence range (in constellations *parallel* and *angle*) or spanned the entire range as well (in constellation *crossing*), representing three distinct ways that the trade-off between defence, growth rate, and plasticity range may play out. For each constellation, we varied the maximum switching rate *χ*_*max*_ over 5 orders of magnitude to investigate the effect of plasticity (Table 1, middle row). This parameter determines how rapidly a species can switch between phenotypes (see “Methods”, “Exchange rates”); higher values indicate faster adaptation. These results were also compared with a non-plastic baseline scenario where both phenotypes of each species are presented but *χ*_*max*_ = 0 (Table 1, upper row), as well as a rigid scenario where the species have only a single phenotype (Table 1, bottom row). All parameters and their values can be found in Supplementary Table S1.

In the following, we give a detailed description of the results for constellation *parallel*, where the autotroph species *A* and *B* have the same defence costs resulting in parallel trade-off lines between defence and growth rate, while varying the level of defence for *A* and varying the plasticity costs for *B* (Table 1, left column). We start with examining patterns for the phenotype biomasses, coexistence and community stability in the non-plastic baseline scenario “*parallel 0*”, and then compare the corresponding scenarios with a low exchange rate (“*parallel 0.01*”) and a high exchange rate (“*parallel 1*”). We next discuss the other two constellations (*crossing* and *angle*, Table 1) more briefly. Finally, we generalize across all scenarios and focus on the coexistence, the degree of maladaptive switching, and the consumer and total autotroph biomasses.

### Non-plastic baseline dynamics: scenario *parallel 0*

In this scenario, four single phenotypes unconnected by exchange compete with each other. Thus, species coexistence here depends entirely on phenotype coexistence: the trade-offs have to be such that for each species, at least one phenotype is a good enough competitor to survive. Which phenotypes survive depend on the two trade-off parameters, defence of the defended phenotype of species *A* (*d*_*Au*_) and plasticity costs for species *B* (*pc*_*B*_), which thus determine whether coexistence is possible.

**Figure 2:**
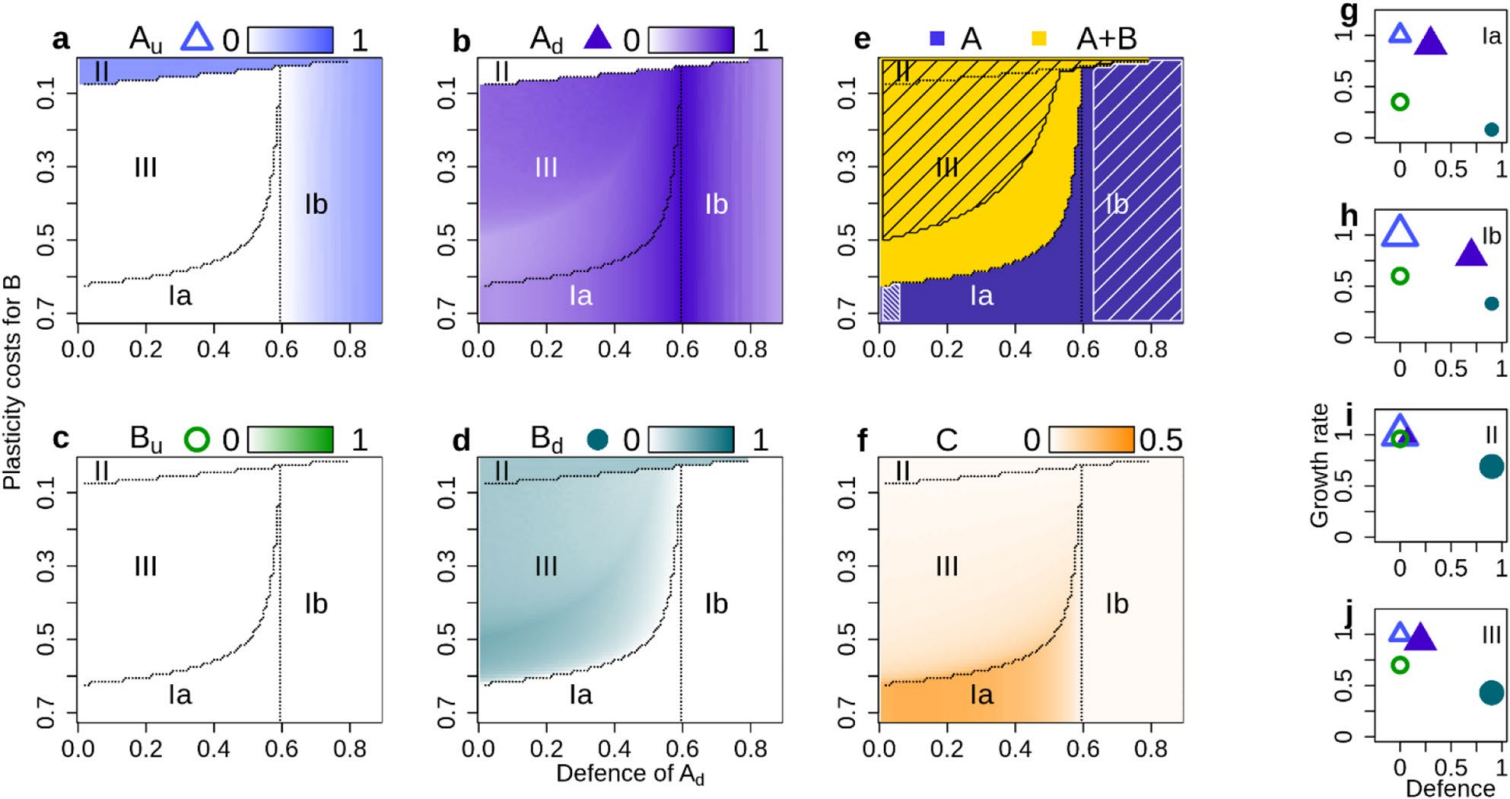
Biomasses, coexistence and trait space for scenario *parallel 0*. Biomasses of the four autotrophic phenotypes (**a**–**d**), their coexistence patterns (**e**), the consumer biomass (**f**) and the autotrophs’ trait values (**g**–**j**) (higher biomasses are shown by darker colours). Lines in (**a**–**f**) separate the regions I–III of different coexistence patterns. Note that in (**a**–**f)**, the y-axis is reversed to show increasing fitness along all axes. An exemplary trait combination for every region is shown in (**g**–j**)**; larger symbols indicate the surviving phenotypes. Shaded areas in (**e**) depict oscillating systems (quarter-lag predator–prey cycles in dense shading, antiphase cycles in loose shading).

The defence costs were kept constant at an intermediate value of 0.3 for both species, resulting in parallel trade-off lines (Table 1, scenario “*parallel 0*”). The undefended phenotype of A, *A*_*u*_, is a growth-specialist with the highest growth rate of all phenotypes. The defended phenotype of the same species, *A*_*d*_, has a defence between 0 and 0.9 and a relatively high growth rate, and can be viewed as a generalist. Species B has variable plasticity costs that lower the growth rate of both phenotypes. The defended phenotype of species B, *B*_*d*_, has the lowest growth rate of all phenotypes but is very well-defended, and thus a defence-specialist. Its undefended phenotype, *B*_*u*_, is as undefended as *A*_*u*_ but has a lower growth rate; it is thus always an inferior competitor and inevitably goes extinct (Fig. 2c).

As *B*_*u*_ never survives, coexistence of the autotroph species requires the survival of defence-specialist *B*_*d*_. *B*_*d*_ can only survive if *A*_*d*_ is not too defended, because *A*_*d*_ has a higher growth rate than *B*_*d*_ and will outcompete *B*_*d*_ in the “defended” niche otherwise (region Ib; Fig. 2d,h). A second criterion is that the plasticity costs for *B* must not be too high, because then the benefits of the defence of *B*_*d*_ no longer outweigh the costs, and it will go extinct even if there are no other highly defended phenotypes around (region Ia; Fig. 2d,g). In the regions where *B*_*d*_ goes extinct, species coexistence is not possible (Fig. 2e). The generalist *A*_*d*_ either survives by itself (region Ia in Fig. 2b,g) if its defence is low to intermediate, or together with the growth-specialist *A*_*u*_ if its defence is high (region Ib in Fig. 2a,b,h). In the regions II and III where *B*_*d*_ survives, it never survives on its own, but always together with one of the phenotypes of *A*. It coexists with the growth-specialist *A*_*u*_ if the plasticity costs are very low (region II in Fig. 2,i), and together with *A*_*d*_ if they are low to intermediate (region III in Fig. 2,j). These two regions do support species coexistence (Fig. 2e).

In three of the four regions (Ib, II and III in Fig. 2f), consumer biomass is low, because the final community always contains a well-defended phenotype (*A*_*d*_ in region Ib, and *B*_*d*_ in regions II and III); the overall level of defence of the community is relatively high in these regions (Supplementary Figure S1). Conversely, consumer biomass is relatively high in region Ia, because the only surviving autotroph phenotype is relatively fast-growing and fairly undefended (Fig. 2f,g). The regions where a well-defended phenotype survives often show antiphase cycles (Ib, II and III in Fig. 2e). These cycles do not occur in the region where only *A*_*d*_ survives (Ia in Fig. 2e); but regular quarter-lag predator–prey cycles can be found here if *A*_*d*_ is almost entirely undefended.

While the community defence (i.e. mean defence of the autotroph community) depends strongly on the coexisting phenotypes, the community growth rate is roughly constant because over the entire trait space, at least one phenotype with a high growth rate always survives (Supplementary Figure S1). The standing variance of the community defence was high when two phenotypes coexist as they occupy different niches along the defence axis (Fig. 2h–j). In contrast, the variance of the community growth rate was very low and almost constant across all regions.

### Effect of phenotypic plasticity

**Figure 3:**
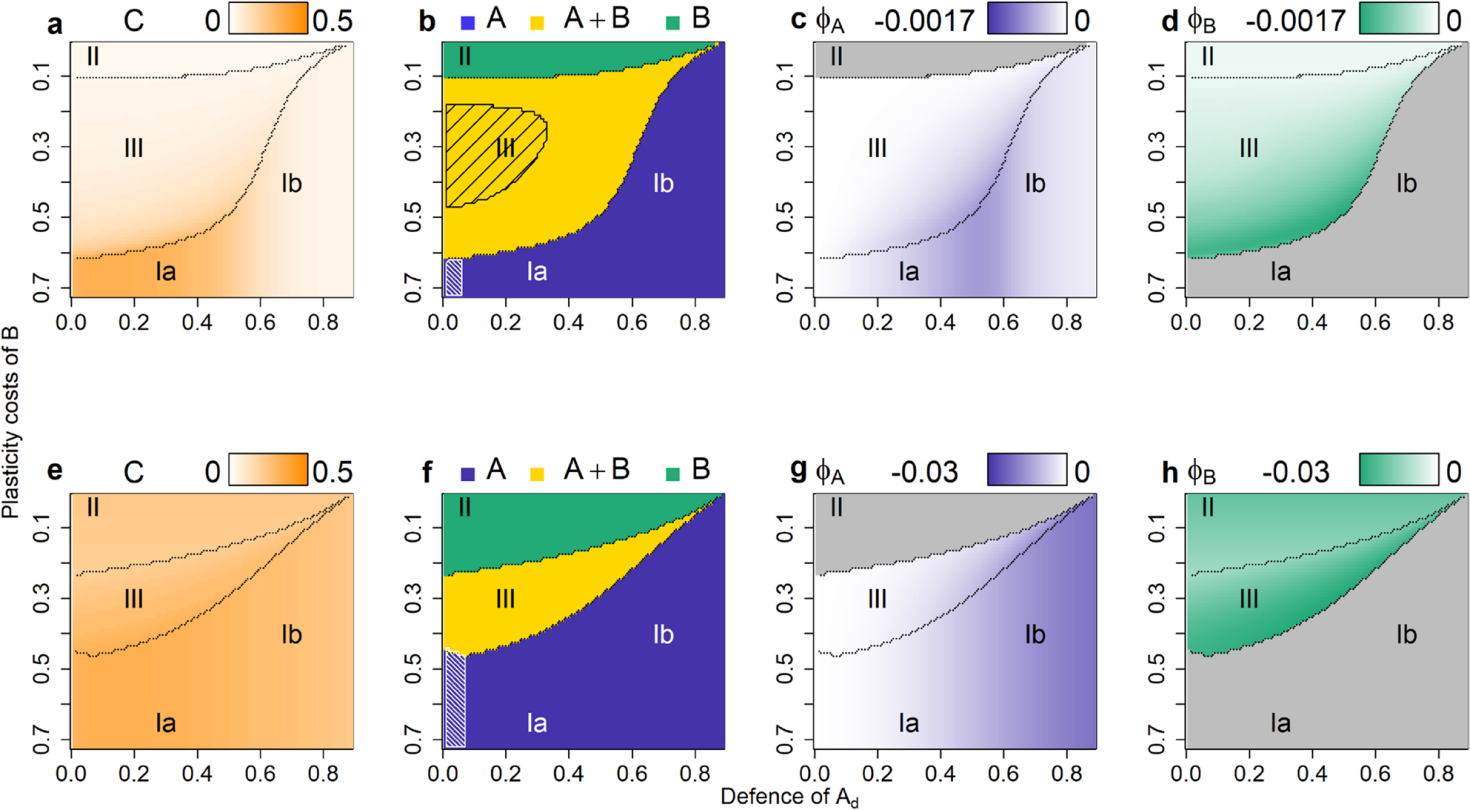
Consumer biomass, autotroph coexistence and maladaptive switching for the scenarios *parallel 0.01* (**a**–**d**) and *parallel 1* (**e**–**h**)*.* Consumer biomass (**a**,**e**), the autotroph coexistence patterns (**b**,**f**), and the autotrophs’ maladaptive switching Φ (**c**,**d**,**g**,**h**) (higher biomasses or more intensive maladaptive switching are shown by darker colours). Lines separate the regions I–III of different autotroph coexistence. The y-axis is reversed to follow the pattern of increasing fitness. Grey areas in (**c**,**d**,**g**,**h**) depict areas where the species was extinct. Shaded areas in b and f depict oscillating systems (quarter-lag predator–prey cycles in dense shading, antiphase cycles in loose shading).

Even a little bit of plasticity in the scenario *parallel 0.01* (*χ*_*max*_ = 0.01) can change the above patterns for coexistence, stability, and average consumer biomass (Fig. 3a–d). While the autotrophs are intuitively expected to benefit from being plastic, the effect of plasticity on consumer biomass always turned out to be positive (Fig. 3a). This may be explained by the fact that switching was always, on average, maladaptive (Fig. 3c,d), measured by the adaptation index Φ (see Eqs. (11–13) in “Methods”). This index combines information on the net “flow” of individuals due to switching (i.e. whether more undefended individuals switch to defended or vice versa) with the fitness difference between the two phenotypes, and thus measures whether overall, more individuals switch from a low-fitness to a high-fitness phenotype (adaptive) or the reverse (maladaptive). This index can approach zero, but is always negative at equilibrium (see Appendix B), indicating maladaptive switching.

The most striking effect of plasticity was on coexistence, which was affected both positively and negatively by plasticity in different regions of the parameter space (Fig. 3b, Supplementary Figure S4a–d). A negative effect on coexistence is seen in region II, where the autotroph species previously coexisted (Fig. 2e), while with plasticity, *B* outcompeted *A* (Fig. 3b). Without plasticity, coexistence was possible in this region because *A*_*u*_ and *B*_*d*_ survived; importantly, *A*_*u*_ outcompeted *B*_*u*_ due to its higher growth rate, even though the difference between their growth rates is very small in this region (Fig. 2i). Plasticity reverses the competitive exclusion pattern between the two undefended phenotypes: *B*_*u*_ receives a constant flow of biomass from the well-defended *B*_*d*_, which compensates for its slightly lower growth rate and allows it to outcompete *A*_*u*_. Thus, coexistence is reduced as a direct consequence of maladaptive switching.

Plasticity can also promote coexistence, as the coexistence region now extends into former region Ib where the generalist *A*_*d*_ is highly defended (Fig. 2b, Supplementary Figure S1a). This is also an effect of maladaptive switching, though in this case the effect is indirect, mediated through the effect of plasticity on consumer biomass. Without plasticity, coexistence was impossible in region Ib because *B*_*d*_ was always outcompeted by *A*_*d*_: even though the latter had a slightly lower level of defence, this was outweighed by its higher growth rate, making *A*_*d*_ the superior competitor over *B*_*d*_. However, plasticity changes this because maladaptive switching increases the consumer biomass, which in turn alters the cost/ benefit balance of defence: *B*_*d*_ derives a stronger benefit from its high level of defence, which now outweighs the cost and allows it to survive. Coexistence through this mechanism is not possible when the plasticity costs for *B* are too high or when *A*_*d*_ is too well-defended, explaining the narrowing of the coexistence “tail” for high defence of *A*_*d*_ (Fig. 3b).

While the patterns of coexistence changed when allowing for plasticity, the patterns in the trait values were nearly indistinguishable from the previous scenario (Supplementary Figure S1, S2). Finally, plasticity had a strong impact on the community dynamics, as most of the antiphase cycles were stabilized (Ib, II, III in Fig. 3b). Their area decreased sharply as these cycles were characterized by asynchronous dynamics between the two prey phenotypes, which were reduced by plasticity. In contrast, the area of the quarter-lag predator–prey cycles remained unaffected by plasticity.

All the above patterns were found to a far stronger degree with a higher amount of plasticity (*χ*_*max*_ = 1; Fig. 3e–h, Supplementary Figure S4e–h). Consumer biomass increased strongly everywhere (cf. Fig. 3a,e), reflecting the strong increase in the degree of maladaptive switching (cf. Fig. 3c,d,g,h). The higher exchange rates led to more synchronization between the phenotypes, extinguishing the antiphase cycles completely (Fig. 3f). It also decreased the biomass of both defended phenotypes (cf. Supplementary Figure S4b,d,f,h). This in turn led to a lower community defence and a higher community growth rate (Supplementary Figure S3) both contributing to a higher consumer biomass. Finally, there was a sharp decrease in the coexistence region for high plasticity (Fig. 3e). Region II, where *B* outcompetes *A* through maladaptive switching, doubled in size due to the much higher degree of maladaptive switching (Fig. 3g,h). Region I, where *A* outcompetes *B*, now also increased, when the level of defence of *A*_*d*_ is relatively low (Fig. 3e). This is again an indirect effect of maladaptive switching causing a strong increase in consumer biomass, affecting the cost/ benefit balance of defence: while *B*_*d*_ derives a strong benefit from its high level of defence, *B*_*u*_ is completely undefended, and is at an extra disadvantage because of its low growth rate. Thus, while *B*_*d*_ would have been able to survive by itself, the high exchange rate causes a strong source-sink dynamic that drives *B* extinct.

### Effect of plasticity in constellations *crossing* and *angle*

In constellation *crossing* the trade-off lines of both species cross in the trait space, as the level of defence is the same for both defended phenotypes; species *B* has a lower growth rate for its undefended phenotype than species *A* due to plasticity costs, while its defence costs are low and thus the growth rate of its defended phenotype is higher than for species *A* (Table 1, Supplementary Figure S5). Without plasticity the crossing trade-off lines lead to coexistence of both species in all simulations as *A*_*u*_ and *B*_*d*_ were always the only survivors, mostly showing antiphase oscillations (Supplementary Figure S5).

**Figure 4:**
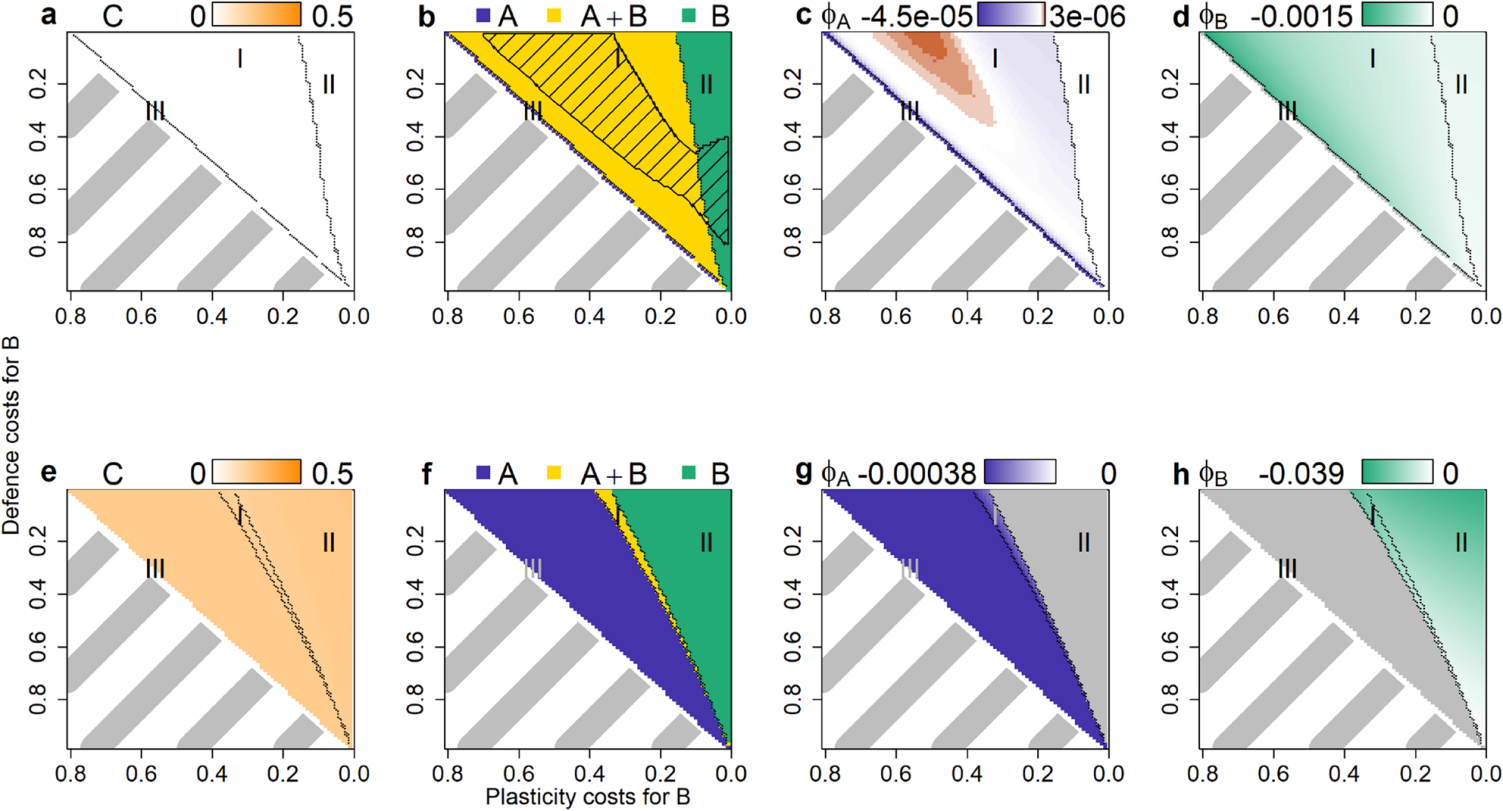
Coexistence and maladaptive switching for scenario *crossing 0.01* (**a**-**d**) and *crossing 1* (**e**–**h**). Consumer biomass (**a**,**e**), the autotroph coexistence patterns (**b**,**f**), and the autotrophs’ maladaptive switching Φ (**c**,**d**,**g**,**h**) (higher biomasses or more intensive maladaptive switching are shown by darker colours). Lines separate the regions I–III of different autotroph coexistence. The x- and y-axis are reversed to follow the pattern of increasing fitness. Shaded areas in (**b**) depict antiphase cycles. Grey areas in (**c**,**d**,**g**,**h**) depict areas where the species was extinct. Shaded grey areas depict areas without simulations (cf. “Methods”). Note that (**c**,**d**,**g**,**h**) have each a different colour scale.

Allowing for phenotypic plasticity has the same results as were observed for constellation *parallel*: consumer biomass sharply increases (Fig. 4a,e); antiphase cycles are dampened or absent; and the area of coexistence decreases (Fig. 4b,f). All these changes are more pronounced for higher exchange rates (cf. Fig. 4a,b,e,f). Again, the biomass of the defended phenotypes decreased for high exchange rates (Supplementary Figure S6). Switching was always maladaptive for high exchange rates (Fig. 4g,h), and mostly maladaptive for low exchange rates (Fig. 4c,d). As was seen for constellation *parallel*, maladaptive switching was the reason for the decrease in coexistence. *B* can outcompete *A* when *B* has low plasticity costs. *B*_*d*_ has a much higher growth rate than *A*_*d*_, while the undefended phenotypes have similar growth rates. The direction of competitive exclusion between *A*_*u*_ and *B*_*u*_ is thus easily reversed by *B*_*d*_ donating biomass to the sink *B*_*u*_, allowing *B* to occupy both niches and outcompete *A* (region II in Fig. 4b,f). The same mechanism happens in reverse for high plasticity and defence costs of *B*: the differences in growth rate for the undefended phenotypes are high, while the defended phenotypes have very similar growth rates. *A*_*u*_ can support *A*_*d*_, and *A* outcompetes *B* (region III in Fig. 4b,f).

In constellation *angle* there are no plasticity costs, and thus the undefended phenotypes *A*_*u*_ and *B*_*u*_ have identical growth rates. The defended phenotypes take the same places in trait space as in the *parallel* constellation: *A*_*d*_ is a generalist, with a lower level of defence and a relatively high growth rate due to low defence costs, whereas *B*_*d*_ is a defence-specialist with a high level of defence but a low growth rate. This leads to the trade-off lines forming an angle (see Table 1). Without phenotypic plasticity, the coexistence patterns are the same as in constellation *parallel*, except that no competitive exclusion occurs between the undefended phenotypes; instead, they (neutrally) coexist in regions Ib, II and III (Supplementary Figure S7; cf. Fig. 2).

**Figure 5:**
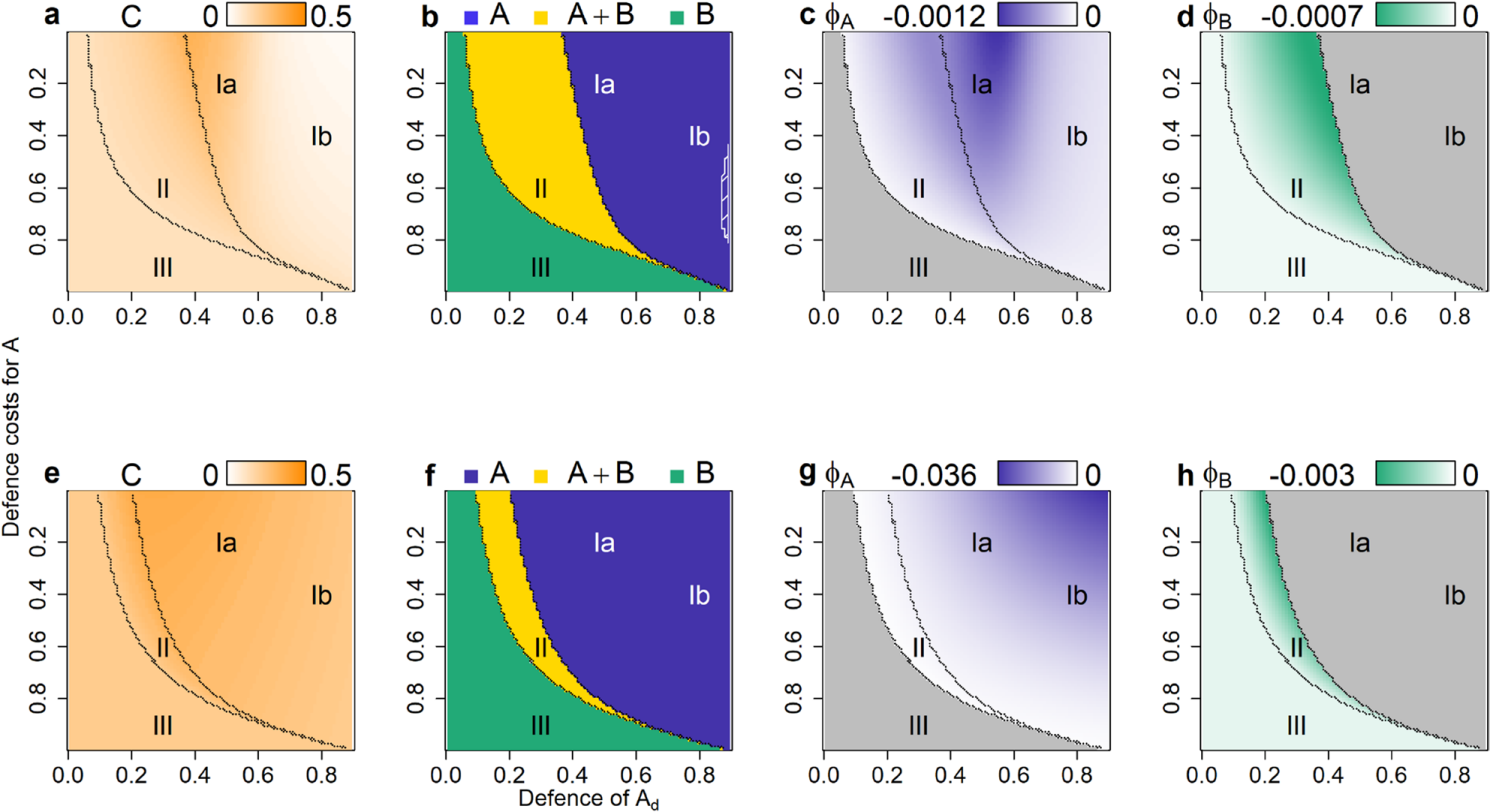
Coexistence and maladaptive switching for scenario *angle 0.01* (**a**–**d**) and *angle 1* (**e**–**h**). Consumer biomass (**a**,**e**), the autotroph coexistence patterns (**b**,**f**), and the autotrophs’ maladaptive switching Φ (**c**,**d**,**g**,**h**) (higher biomasses or more intensive maladaptive switching are shown by darker colours). Lines separate the regions I–III of different autotroph coexistence. The y-axis is reversed to follow the pattern of increasing fitness. Shaded areas in (**b**) depict antiphase cycles. Grey areas in (**c**,**d**,**g**,**h**) depict areas where the species was extinct. Note that (**c**,**d**,**g**,**h**) have each a different colour scale.

With plasticity, neutral coexistence vanished: the defended phenotype that survived (*A*_*d*_ in region Ib, *B*_*d*_ in region III) could support the undefended phenotype of its own species, driving the other species extinct (Fig. 5b,f). As in the other constellations, the area of coexistence and the biomasses of the defended phenotypes decreased and antiphase cycles vanished with increasing *χ*_*max*_ (Fig. 5b,f, Supplementary Figure S8), while maladaptive switching and the consumer biomass increased (Fig. 5).

### General results

**Figure 6:**
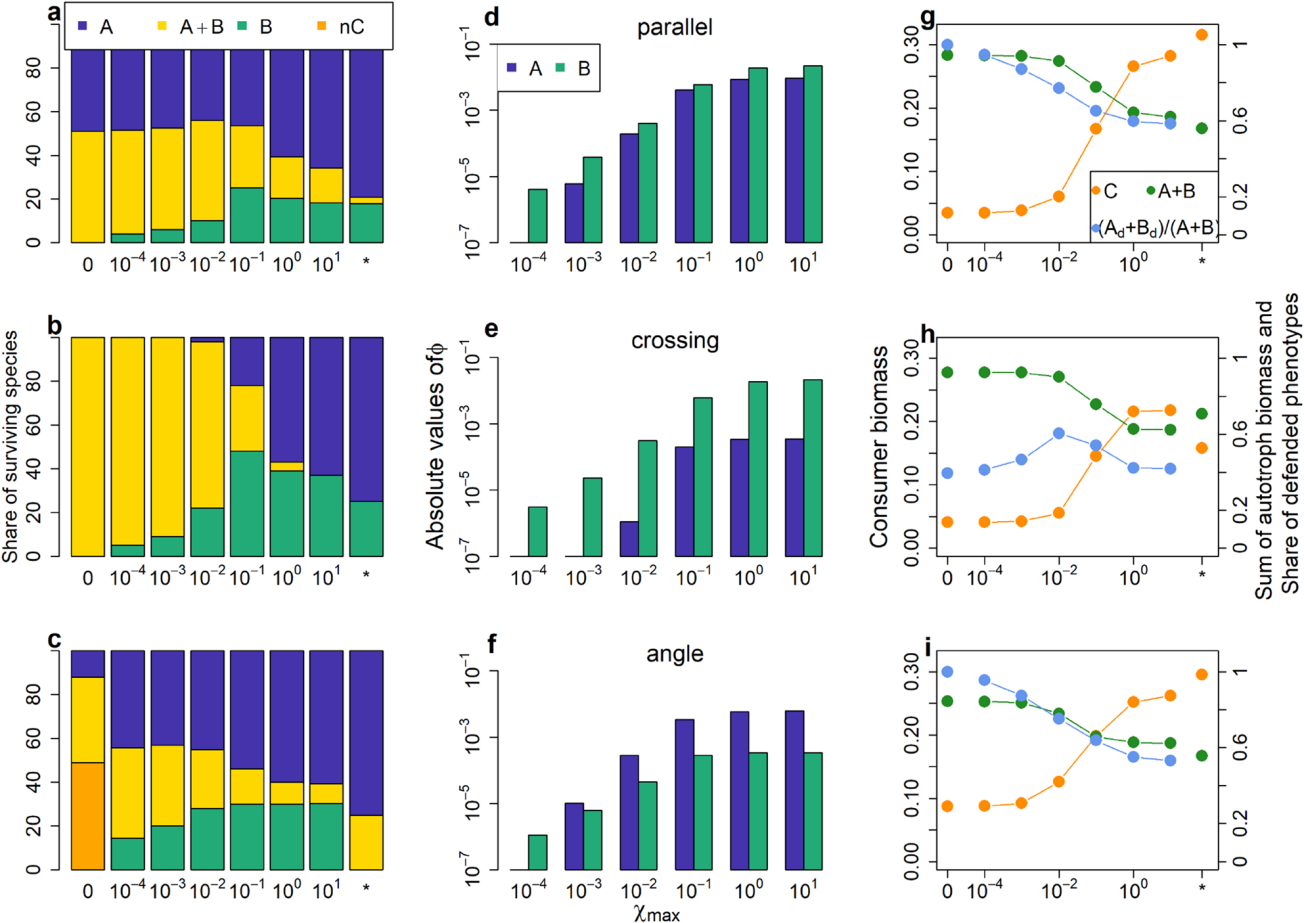
General patterns for coexistence, maladapative switching and biomasses. Share of surviving species in percent (*A*, *B*, coexistence or neutral coexistence) (**a**–**c**), median absolute value of maladaptive switching Φ (**d**–**f**) and median of total autotroph biomass (*A* + *B*), median consumer biomass *C* and share of defended phenotypes ((*A*_*d*_ + *B*_*d*_)/(*A* + *B*)) (**g**–**i**) for the three constellations and increasing maximum exchange rates χ_max_. χ_max_ = 0 denotes the non-plastic scenarios; *denotes the rigid scenarios. Maladaptive switching and the share of defended phenotypes do not apply for the rigid scenarios.

As plasticity had very similar effects across all three constellations, we here generalize our results: we compare the three constellations for exchange rates over 5 orders of magnitude, as well as the non-plastic scenario and the rigid scenario (Table 1). That is, all simulations from one scenario (e.g. *parallel 0*) were summarized into one bar respective point in Fig. 6.

For all constellations, the fraction of simulation runs leading to coexistence was highest in the non-plastic scenario and decreased with increasing *χ*_*max*_ (Fig. 6a–c). In constellation *parallel* the share of coexistence for increasing *χ*_*max*_ continuously decreased from 51 to 3% (Fig. 6a). In *crossing*, the share decreased from full to no coexistence (Fig. 6b). In *angle*, the share of coexistence was 88% in the non-plastic scenario when taking also neutral coexistence into account (Fig. 6c). Its share decreased to 9% for a *χ*_*max*_ of 10 and increased again to 25% for the rigid scenario. Maladaptive switching increased for both species and all constellations for increasing *χ*_*max*_ (Fig. 6d–f). The increased plasticity led to a lower total autotroph biomass and a lower share of defended phenotypes (Fig. 6g–i), which resulted in higher consumer biomass (Fig. 6g–i).

Interestingly, and counterintuitively, the above patterns show that increasing the speed of plasticity (by increasing *χ*_*max*_) makes the system behave more like the rigid system. The coexistence patterns in scenarios with high *χ*_*max*_ approach those of the rigid scenarios in two of the constellations (Fig. 6a,b). Similarly, the total autotroph and consumer biomasses approach the ones in the rigid scenarios (Fig. 6g–i). Thus, we found the higher *χ*_*max*_ make the autotrophs not more adaptive, but behave more like non-adaptive species.

## Discussion

To understand the consequences of phenotypic plasticity, including the consequences of maladaptive switching, we investigated three trade-off constellations in a small food web of two plastic autotrophs and a shared consumer across different levels of plasticity. All constellations showed very consistent patterns: a higher speed of adaptation stabilized the dynamics, decreased the area of autotroph coexistence, increased the degree of maladaptive switching, and thus lowered total autotroph biomass and increased consumer biomass. It is well established that plasticity leads to stabilization^2–5,28^, but the other patterns are more surprising. Most importantly, contrary to the intuitive expectation that plasticity in the defence of its prey is disadvantageous for a predator, as the prey can switch between undefended and defended phenotypes, we find it is beneficial for the predator as maladaptive switching enhances the biomass of its undefended prey.

Most of the patterns we found depend strongly on the result that, in the long term, plasticity results in maladaptive switching between phenotypes. It is important to note that this does not necessarily hold in the short term, because whether switching is adaptive or maladaptive depends very strongly on whether there is temporal variation in predation risk (see Supplementary Information B, Fig. S10). As long as the environment is variable, plastic species benefit from being able to quickly defend against predators when predation pressure is high and save these costs for their defence when predation pressure is low. On the long term, however, plasticity stabilizes the predator–prey dynamics^2,28^, and thus plastic species remove their own advantage arising when living in a variable environment in which they have a competitive advantage^2^. The short-term benefit of switching then is replaced by a long-term disadvantage, as switching is always maladaptive at a stable equilibrium (see Supplementary Information B for a mathematical derivation of this result).

To explain intuitively why switching is maladaptive at a stable equilibrium, we start here by considering a simplified non-plastic scenario: one autotroph with an undefended and defended phenotype *A*_*u*_ and *A*_*d*_ and one consumer. In this case, eventually *A*_*u*_ and *A*_*d*_ settle at an equilibrium state where they have equal fitness^29^ (provided that the balance between costs and benefits of defence enables their stable coexistence). At e.g. low defence costs, the equilibrium biomass of the defended phenotype will be high while those of the undefended phenotype and the consumer will be low. But if the autotrophs can switch, this picture changes. This would not be the case if individuals had perfect knowledge of the exact costs and benefits associated with expressing the defended phenotype at each point in time. However, expression of inducible defences typically relies on interpreting environmental cues (e.g. kairomone concentration informing about the density of predators), which have a strong correlation with the fitness effects of switching, but still give only partial information. In our model we assume that autotrophs base their switching decisions on consumer density *C*: if this is higher than a threshold value (*C* > C*), individuals are more likely to switch from undefended to defended, and the reverse is true if *C* < C*. In the hypothetical example described above, this means that due to the low predation pressure arising from the low consumer biomass due to a dominance of defended prey, the switching rate from the defended towards the undefended phenotypes *χ*_*d*_ is high (cf. Fig. 1). Together with the higher biomass of *A*_*d*_ this results in a net biomass flow towards the undefended phenotype.

Thus, due to switching, the phenotypes are pushed off their original equilibrium biomasses: *A*_*u*_ increases and *A*_*d*_ decreases, which in turn results in an increased consumer biomass. This increases the benefit of defence for *A*_*d*_, causing selection to push for an increase in the relative share of *A*_*d*_ while plastic switching continues to push in the opposite direction. Eventually *A*_*u*_ and *A*_*d*_ reach a new equilibrium where selection and switching balance each other out. Since selection is always adaptive (i.e. increasing the frequency of the high-fitness phenotype), and switching always acts to oppose selection at this equilibrium, switching is maladaptive (see Supplementary Information B). Higher switching rates exacerbate this process, and the phenotypes are pushed further away from their original equilibrium leading to a higher degree of maladaptive switching. As the switching rates increase, the shares of defended and undefended phenotypes approach 50:50 (Fig. 6g–i), and the system behaves more and more like the “rigid” baseline where each species has only a single phenotype and thus cannot adapt at all. Thus, counterintuitively, high switching rates appear to make the autotrophs less adaptive rather than more adaptive, resulting in a higher biomass for undefended phenotypes and thus a higher consumer biomass.

One parameter that has a strong impact on the degree of maladaptive switching is the threshold C*: if this threshold is set very high or very low, autotrophs will either start defending too early, when the costs still outweigh the benefits, or remain undefended too long. By choosing the value of C* based on the range of consumer densities the autotrophs are likely to encounter (see “Methods” for details) we aimed for switching to be mostly adaptive, but avoiding maladaptive switching entirely is impossible when switching decisions are based on a single environmental cue. There is evidence that some species can detect additional cues, such as conspecific density^19,30^, or use alarm cues which measure predation risk more directly^31^, both of which would probably reduce the degree of maladaptive switching. Another important factor is the degree of temporal variation in predation risk, as the argumentation in the previous paragraphs only holds under a stable equilibrium. In accordance with a previous study^23^, we found that switching can be adaptive in the long run if there are ongoing oscillations (Fig. 4c). We rarely found this as a long-term outcome given the stabilizing effect of plasticity. However, as natural food web dynamics are complex and include many species and are subject to temporal variation in abiotic conditions, the stabilizing impact of plasticity may be counteracted under natural conditions. Although predator oscillations have been shown to be dampened by inducible defences in situ^3^, they may not disappear entirely, and the degree of maladaptive switching may therefore be less severe in nature than found in our model.

In addition to affecting stability and consumer biomass, plasticity also had consequences for competition, and thereby for coexistence of the two autotroph species. Without plasticity, species coexistence is determined by which phenotypes can coexist, which is determined by their locations in trait space^29^ (see Supplementary Figure S9). Plasticity can change these coexistence patterns, allowing phenotypes to survive where they would have gone extinct without plasticity, or vice versa. A clear example is the survival of *A*_*u*_ and the extinction of *B*_*u*_ without plasticity (region II in Fig. 2), a pattern which was reversed by even a small amount of plasticity (region II in Fig. 3) through the source-sink dynamics generated by maladaptive switching. Such effects on survival are particularly likely to happen when the fitness difference between two competing phenotypes is small, as was the case between *A*_*u*_ and *B*_*u*_ in region II (Fig. 2i). Without plasticity *A*_*u*_ could always outcompete *B*_*u*_, but because its growth rate was only slightly higher than that of *B*_*u*_ and *B*_*d*_ is a good competitor with high defence and only low plasticity costs, the biomass flow caused by plasticity could easily overwhelm this and allow *B*_*u*_ to survive instead. This allows one of the autotroph species to completely outcompete the other one under conditions when two non-plastic species coexisted. This result is very similar to the effect of dispersal in metacommunities, where source-sink dynamics between patches can change coexistence and lead to the extinction of the locally superior competitor^32,33^.

Plasticity can also affect coexistence indirectly: it causes an increase in consumer biomass, which affects the fitness of all phenotypes (increasing the fitness of well-defended ones and decreasing the fitness of undefended ones), which can in turn alter coexistence, sometimes increasing the coexistence range (Fig. 3b vs Fig. 2e), but more often decreasing it (Fig. 3f vs. Fig. 3b). Thus, the combined effect of inducible defences on coexistence is highly complex, but overall it reduces the potential for competitive coexistence, and more strongly so for faster switching. While the notion that phenotypic plasticity may hinder coexistence is quite well established^34^, current theory focuses on traits that are directly involved in competition and resource uptake, whereas in our system the effects of phenotypic plasticity are also mediated through the interaction between autotrophs and their consumer. Despite this difference, our conclusions are largely the same: inducible defences impact coexistence through their effect on niche differences and fitness differences^34^. Niche differences are reduced because the species can occupy broader niches through switching, while fitness differences are affected (sometimes increased and sometimes decreased) by maladaptive switching, as well as by the impact on consumer biomass. In addition, the source-sink dynamic arising from maladaptive switching equalizes phenotype biomasses at high switching rates, similar to how dispersal in metacommunities decreases inter-patch diversity due to homogenization^35^, which reduces niche differences between the competing species further and further until their coexistence becomes impossible.

To represent inducible defences in our model, we used switching functions, which are commonly used for binary defence mechanisms where individuals can switch between discrete undefended and defended phenotypes^27^. Alternatively inducible defences can be modelled using the fitness gradient approach or the optimal gradient approach. Since the dynamical consequences of inducible defences can depend on the approach used^27^, the impact on maladaptive switching, consumer biomass and coexistence may depend on the modelling approach as well. However, directly measuring maladaptive switching as a consequence of plasticity is only possible with the switching function approach, since only this approach explicitly incorporates individual switching decisions. In contrast, the fitness gradient and optimal trait are phenomenological approaches, which consider the average trait in the population instead of modelling individual behaviour, thus making it impossible to measure maladaptive switching. The commonness of these approaches^27^, and their underlying assumption that plastic decisions increase fitness, may have contributed to the way that maladaptive switching has long been unnoticed in theoretical studies on the ecological consequences of inducible defences.

Overall, our results show very consistent patterns: a higher switching rate stabilized the dynamics, decreased the area of coexistence for both autotrophic species and increased the degree of maladaptive switching and the consumer biomass. Thus, we conclude that inducible defences can be a double-edged sword: a plastic species may outcompete its competitors via source-sink dynamics, but the negative impact of maladaptive switching can also lower its biomass or even drive it extinct. Plasticity can also have both negative and positive impacts on the food web level: when species are outcompeted, the food web diversity decreases; but the system is stabilized which may prevent further species loss due to strong oscillations. Moreover, energy transfer to higher trophic levels is enhanced as more undefended prey are available to the consumer due to maladaptive switching. Maladaptive switching may also prevent plastic species from becoming Darwinian Demons counteracting the extinction of other species. Finally, maladaptive switching may also contribute to the fact that plasticity is not universal, despite its seemingly obvious advantages in rapidly changing environments prevailing almost everywhere. Overall, the striking impact of maladaptive switching on species and food web dynamics indicates that this mechanism may be of critical importance for large-scale effects of inducible defences. To what extent these effects can be generalized to more trophic levels and larger food webs under externally forced environmental conditions will be an important subject for future investigations.

## Methods

### Food web structure

We consider a food web with two plastic autotroph species *A* and *B*, having each an undefended (*A*_*u*_ resp. *B*_*u*_) and a defended (*A*_*d*_ resp. *B*_*d*_) phenotype. The autotrophs compete for the same resources (modelled as a shared carrying capacity) and are predated on by a joint consumer *C* (Fig. 1a).

Phenotype *j* of autotroph species *i* grows logistically with a growth rate *r*_*ij*_ and a carrying capacity *K*_*ij*_ (Eqs. 1–4). It is grazed by the consumer following a Holling type II functional response with attack rate *a*_*ij*_ and handling time *h*. Switching between two phenotypes of the same species is represented by the exchange rates *χ*_*j*_. The consumer *C* converts the captured biomass with a conversion efficiency *ε* into own biomass, and dies with a death rate *δ* (Eq. 5).

Defence is modelled as a binary trait: a species expresses either an undefended or a defended phenotype, e.g. either it grows spines or does not. A second parameter is the defence value, analogous to the spine length of an algal species protecting algae less or more against their predators. The undefended phenotypes have a defence value of 0, (i.e. no defence), while the defended ones have a defence value *d*_*ij*_ between 0.01 and 0.9, meaning 1–90% of the biomass cannot be consumed by the consumer. Defence is modelled as a pre-attack defence against the consumer as it scales the maximum attack rate *a*^36^ (Eq. 7).

1 $${\dot{\text{A}}}_{u} = { }\underbrace {{r_{Au} \left( {1 - \frac{{A_{u} + A_{d} + B_{u} + B_{d} }}{{ K_{Au} }}} \right)A_{u} }}_{{\text{per capita growth }}} - \underbrace {{\frac{{a_{Au} A_{u} C}}{{1 + h\left( {a_{Au} A_{u} + a_{Ad} A_{d} + a_{Bu} B_{u} + a_{Bd} B_{d} } \right)}}}}_{{{\text{mortality}}}} - \underbrace {{\chi_{u} A_{u} + \chi_{d} A_{d} }}_{{{\text{exchange}}}}$$

2 $${\dot{\text{A}}}_{d} = { }\underbrace {{r_{Ad} \left( {1 - \frac{{A_{u} + A_{d} + B_{u} + B_{d} }}{{ K_{Ad} }}} \right)A_{d} }}_{{\text{per capita growth }}} - \underbrace {{\frac{{a_{Ad} A_{d} C}}{{1 + h\left( {a_{Au} A_{u} + a_{Ad} A_{d} + a_{Bu} B_{u} + a_{Bd} B_{d} } \right)}}}}_{{{\text{mortality}}}} + \underbrace {{\chi_{u} A_{u} - \chi_{d} A_{d} }}_{{{\text{exchange}}}}$$

3 $${\dot{\text{B}}}_{u} = { }\underbrace {{r_{Bu} \left( {1 - \frac{{A_{u} + A_{d} + B_{u} + B_{d} }}{{ K_{Bu} }}} \right)B_{u} }}_{{\text{per capita growth }}} - \underbrace {{\frac{{a_{Bu} B_{u} C}}{{1 + h\left( {a_{Au} A_{u} + a_{Ad} A_{d} + a_{Bu} B_{u} + a_{Bd} B_{d} } \right)}}}}_{{{\text{mortality}}}} - \underbrace {{\chi_{u} B_{u} + \chi_{d} B_{d} }}_{{{\text{exchange}}}}$$

4 $${\dot{\text{B}}}_{d} = { }\underbrace {{r_{Bd} \left( {1 - \frac{{A_{u} + A_{d} + B_{u} + B_{d} }}{{ K_{Bd} }}} \right)B_{d} }}_{{\text{per capita growth }}} - \underbrace {{\frac{{a_{Bd} B_{d} C}}{{1 + h\left( {a_{Au} A_{u} + a_{Ad} A_{d} + a_{Bu} B_{u} + a_{Bd} B_{d} } \right)}}}}_{{{\text{mortality}}}} + \underbrace {{\chi_{u} B_{u} - \chi_{d} B_{d} }}_{{{\text{exchange}}}}$$

5 $$\dot{C} =\left(\frac{\varepsilon ({{a}_{Au}A}_{u}+{a}_{Ad}{A}_{d}+{{a}_{Bu}B}_{u}+{{a}_{Bd}B}_{d})}{1+h({{a}_{Au}A}_{u}+{a}_{Ad}{A}_{d}+{{a}_{Bu}B}_{u}+{{a}_{Bd}B}_{d})}-\delta \right)C$$

6 $${r}_{ij}=r {(1-pc}_{i}-{dc}_{i} {d}_{ij}), i\in \{A,B\},j\in \{u,d\}$$

7 $${a}_{ij}=a (1-{d}_{ij}), i\in \{A,B\},j\in \{u,d\}$$

8 $${K}_{ij}=K (1-0.1{pc}_{i}-{0.1dc}_{i} {d}_{ij}), i\in \{A,B\},j\in \{u,d\}$$

9 $${\chi }_{u}= \frac{{\chi }_{max}}{1+{e}^{b\left({C}^{*}-C\right)}}$$

10 $${\chi }_{d}= {\chi }_{max}(1-\frac{1}{1+{e}^{b\left({C}^{*}-C\right)}})$$

### Trade-offs

Trade-offs define the possible combinations of trait values a species can have under the given biological and energetic constraints, i.e. a species cannot optimize all its traits (defence *d*_*ij*_ and maximum growth rate *r*_*ij*_) simultaneously. An illustration of the possible trait combinations is shown in Fig. 1b. The phenotypes’ positions in the trait space of *r*_*ij*_ and *d*_*ij*_ are defined by three trade-off properties:

- *d*_*ij*_ determines the level of defence.
- *dc*_*i*_ (defence costs) determine how costly it is to produce the defended phenotype; we assume that defended phenotypes always have a lower growth rate than their undefended counterparts of the same species due to resources needed to express the defence. Reflecting this, dc_*i*_ defines the slope of the trade-off line between the growth rate *r*_*ij*_ and the defence *d*_*ij*_, (Fig. 1b, Eq. 6).
- *pc*_*i*_ (plasticity costs) determines the costliness of having a high degree of plasticity, in our model corresponding to having a broad range of defence (Fig. 1b). This is implemented as a reduced growth rate for both phenotypes of a species, e.g. genetic information to sense predator abundance and put the defence into practice that has to be carried along whether the phenotype produces the toxins or not (Fig. 1b, Eq. 6).

These three key parameters define the phenotypes’ location along the growth rate axis and multiplied with the maximum growth rate *r* they define the growth rate *r*_*ij*_ (Eq. 6). The autotrophs face an additional trade-off between *d*_*i*_ and the capacity *K*_*ij*_ (Eq. 8). This trade-off is implemented in the same way as the one between *d*_*ij*_ and *r*_*ij*_, but with only 10% of the strength. This second trade-off represents the costs of defence that manifest when resources are scarce (see e.g. ^14,37–39^), e.g. a thick cell wall as defence which reduces the nutrient uptake, while the trade-off between *r*_*ij*_ and *d*_*ij*_ is the dominant one under rich resource conditions.

### Exchange rates

Inducible defences with binary traits are well represented by switching functions^27^ defining the exchange rate *χ*_*j*_ between the two phenotypes, which depends on the consumer biomass to represent grazing pressure. The exchange rate *χ*_*u*_ defines the switching from the undefended to the defended phenotypes, which increases with increasing consumer biomass as the defence is needed, while the exchange rate towards the undefended phenotype *χ*_*d*_ decreases with consumer biomass (Eqs. 9–10, Fig. 1c). The maximum exchange rate *χ*_*max*_ scales the exchange rates, and the steepness of the switching function is determined by *b*. The inflection point C* denotes the point at which both exchange rates are equal (Fig. 1c). To ensure the inflection point has an ecologically reasonable value, we set it to half of the maximum consumer density in a simulation in which *b* is set to zero, thus having a constant exchange rate of *χ*_*max*_/2. Other model parameters can be found in Supplementary Table S1.

### Scenarios

Varying two of the three trade-off properties (defence, defence costs and plasticity costs) and keeping the third one constant leads to three constellations *parallel*, *crossing*, and *angle* (Table 1). In all three, the autotrophic species *B* spans the entire defence range, i.e. it has a completely undefended phenotype *B*_*u*_ and a maximally defended phenotype *B*_*d*_. Species *A* either has a more limited defence range (in constellations *parallel* and *angle*) or spans the entire range as well (in constellation *crossing*), representing three distinct ways that the trade-off between defence and growth may play out.

In constellation *parallel* both species have the same defence costs leading to parallel trade-off lines between defence and growth rate; *A* has a higher growth rate, but a lower plasticity range, whereas *B* has a lower growth rate due to plasticity costs, but a highly defended phenotype (Table 1, left column). Such a constellation was observed for *Daphnia pulex* clones^40^ and for *Brachionus* species^41^.

In constellation *crossing* the level of defence is the same for both defended phenotypes. The growth rate of *B*_*u*_ is lower than that of *A*_*u*_ due to plasticity costs, while the defence costs of *B* are low and thus the growth rate of *B*_*d*_ is relatively high. In contrast, *A* has a fast-growing undefended phenotype and high defence costs leading to a slow-growing defended phenotype. The trade-off lines of both species thus cross in the trait space (Table 1, middle column).

In constellation *angle* there are no plasticity costs^17^, and thus the undefended phenotypes have identical growth rates. *A*_*d*_ has a high growth rate due to low defence costs but a smaller plasticity range, whereas *B* has high defence costs for its highly defended phenotype leading to a slow-growing but well defended phenotype. Due to the identical growth rate of the undefended phenotypes, both trade-off lines form an angle (Table 1, right column).

To investigate the effect of the speed of adaptation, we varied the maximum exchange rate *χ*_*max*_ between 10^–4^ and 10^1^ in 6 logarithmic steps. These simulations were also compared to a non-plastic baseline scenario where the exchange rates were set to zero, as well as to a completely non-adaptive scenario where each autotrophic species had only one intermediate phenotype (Table 1). This yielded 24 scenarios (three constellations *parallel*, *crossing*, *angle* with eight levels of adaptiveness).

For each simulation run, the traits of all phenotypes were fixed. Phenotypic plasticity via the exchange rate was thus the only possibility for the species to adapt. For each constellation, two of the three properties were varied in 89 or 99 steps leading to 7921 or 8811 simulation runs per scenario to ensure a wide trait range being simulated. In constellation *crossing*, the combination of high plasticity and defence costs for B reduces the growth rate of its defended phenotype below zero; these simulations were excluded from the analysis (shaded grey areas in Fig. 4).

### Modelling details and analysis

Each simulation was run for 100,000 time steps. The time series of the last 10,000 time steps were used for all calculations. For each simulation, we calculated the mean biomasses of each autotrophic phenotype and the consumer, their extinction status (a phenotype was regarded as extinct if its mean biomass was below 10^–6^), autotroph coexistence, and the stability of community dynamics. The two autotrophs coexist if at least one phenotype of each species persists at the end of the simulation. The system was regarded as stable if the coefficient of variation, calculated for the always persistent consumer, was below 0.1. In addition to regular quarter-lag predator–prey cycles, we also found antiphase cycles^38,42^ due to phenotype sorting. R (version 4.0.0) and the package deSolve was used for all simulations and the analysis.

### Maladaptive switching

Whether switching is adaptive or maladaptive at any given point in time depends on whether more individuals switch from the lower-fitness phenotype to the higher-fitness phenotype (adaptive) or the reverse (maladaptive). A measure for adaptiveness or maladaptiveness in switching thus needs to contain two elements: the net flow between the phenotypes, and the fitness difference between them. The relative net flow can, exemplary here for species *A*, be defined as:

11 $${{\Delta }_{\chi }}_{A}=\frac{{A}_{u}{\chi }_{u}-{A}_{d}{\chi }_{d}}{{A}_{u}+{A}_{d}}$$

Note that ${\Delta }_{\chi A}$ measures the fraction of the autotroph species *A* that switches from the undefended to the defended state. When the undefended phenotype is dominant (and *χ*_*u*_ is not too low due to very low consumer biomasses), ${\Delta }_{\chi A}$ is positive and there is a relative net flow to the defended phenotype. In contrast, when the defended phenotype dominates (and *χ*_*d*_ is not too low due to very high consumer biomasses), ${\Delta }_{\chi A}$ is negative resulting in a relative net flow to the undefended phenotype.

The fitness difference is defined as:

12 $${\Delta }_{{F}_{A}}={F}_{Ad}-{F}_{Au},$$

where *F*_*Au*_ and *F*_*Ad*_ represent the per capita net growth rates of the undefended and defended phenotypes, respectively (that is, the difference between their per capita growth and mortality terms; see Eqs. (1,4)).

If ${{\Delta }_{\chi }}_{A}$ and ${\Delta }_{{F}_{A}}$ are both positive, there is net flow from undefended to defended phenotypes and defended phenotypes have higher fitness; switching is thus adaptive. The same is true if both terms are negative. On the other hand, if one of these two terms is positive and the other is negative, however, there is maladaptive switching: more individuals switch from the higher-fitness phenotype to the lower-fitness one than vice versa. Thus measuring adaptiveness can be done by multiplying these two terms together:

13 $${\Phi }_{A}={{\Delta }_{\chi }}_{A}\cdot {\Delta }_{{F}_{A}}$$

The interpretation of this measure is straightforward: switching is adaptive when Φ > 0 and maladaptive when Φ < 0, and more strongly so for larger absolute values of Φ.

## Supplementary Information

Supplementary Information.
