# Supplementary material for: The double-edged sword of inducible defences: costs and benefits of maladaptive switching from the individual to the community level
Source: Sci Rep. 2022 Jun 20;12:10344. doi: 10.1038/s41598-022-13895-7 (PMC9209413; doi:10.1038/s41598-022-13895-7)
Supplement: Supplementary file 1 — Supplementary Information. [file 41598_2022_13895_MOESM1_ESM.pdf]

The double-edged sword of inducible defences: costs and benefits of maladaptive switching from the individual to the community level

Nadja J. Kath, Ursula Gaedke, Ellen van Velzen

Supplementary Information A: supporting table and figures

Parameter

Table S1 : Model parameters. Standard parameters in the model, their abbreviation, unit, value, and reference.

Name	Unit	Range	Meaning
r	$[\frac{1}{d}]$	1	Maximum growth rate of autotrophs
pc_i	[-]	0.01-1	Plasticity costs
K	$[\frac{mg\ C}{l}]$	1	Maximum carrying capacity
dc_i	[-]	0.01-1	Defence costs
a	$[\frac{1}{d}\frac{l}{mg\ C}]$	6	Maximum attack rate
d_{ij}	[-]	0-0.9	Defence, i.e. reduction of attack rate of consumer on A_{ij}
h	[d]	1	Handling time
χ_{max}	$[\frac{1}{d}]$	0-10 across 5 orders of magnitude	Maximum exchange rate between undefended and defended phenotype of a species
b	$[\frac{l}{mg\ C}]$	10	Shape parameter for the exchange function
C^*	$[\frac{mg\ C}{l}]$	0.251	Half of maximum consumer density when b=0
ε	[-]	0.3	Conversion efficiency
δ	$[\frac{1}{d}]$	0.21	Consumer death rate

Traits

We measured the community traits by calculating the weighted mean and weighted variance of the growth rate and the defence. The mean community trait $\bar{\tau}$ was defined as the biomass weighted mean of the trait values of the individual phenotypes τ_{ij}: $\bar{\tau} = \frac{1}{(A_u+A_d+B_u+B_d)}(A_u\tau_{Au} + A_d\tau_{Ad} + B_u\tau_{Bu} + B_d\tau_{Bd})$. The community variance was defined as weighted variance $var(\tau) = \frac{1}{3(A_u+A_d+B_u+B_d)}(A_u(\tau_{Au} - \bar{\tau})^2 + A_d(\tau_{Ad} - \bar{\tau})^2 + B_u(\tau_{Bu} - \bar{\tau})^2 + B_d(\tau_{Bd} - \bar{\tau})^2)$.

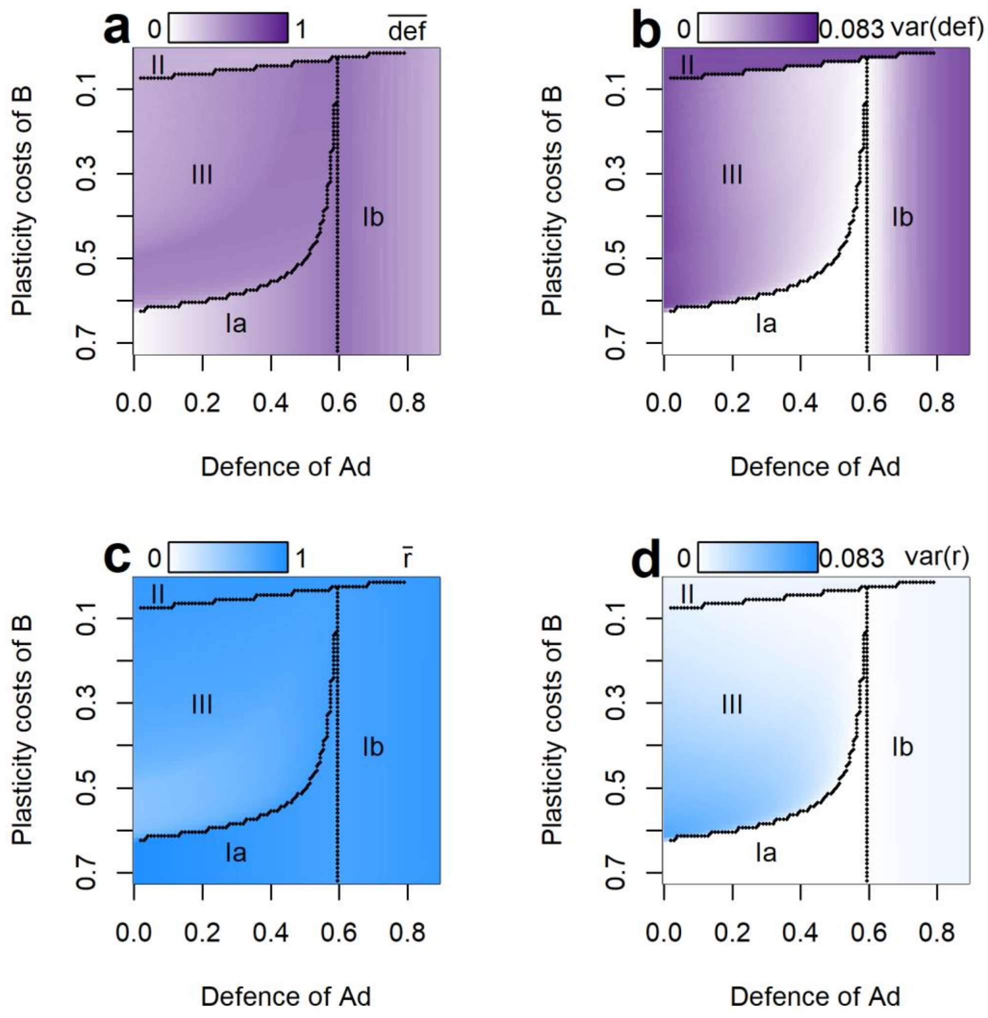

Figure S1: Trait values of the autotroph community for scenario *parallel 0*. Community mean defence (a), variance of the defence (b), community mean growth rate (c) and variance of the growth rate (d). Lines separate the regions I-III of different autotroph coexistence.

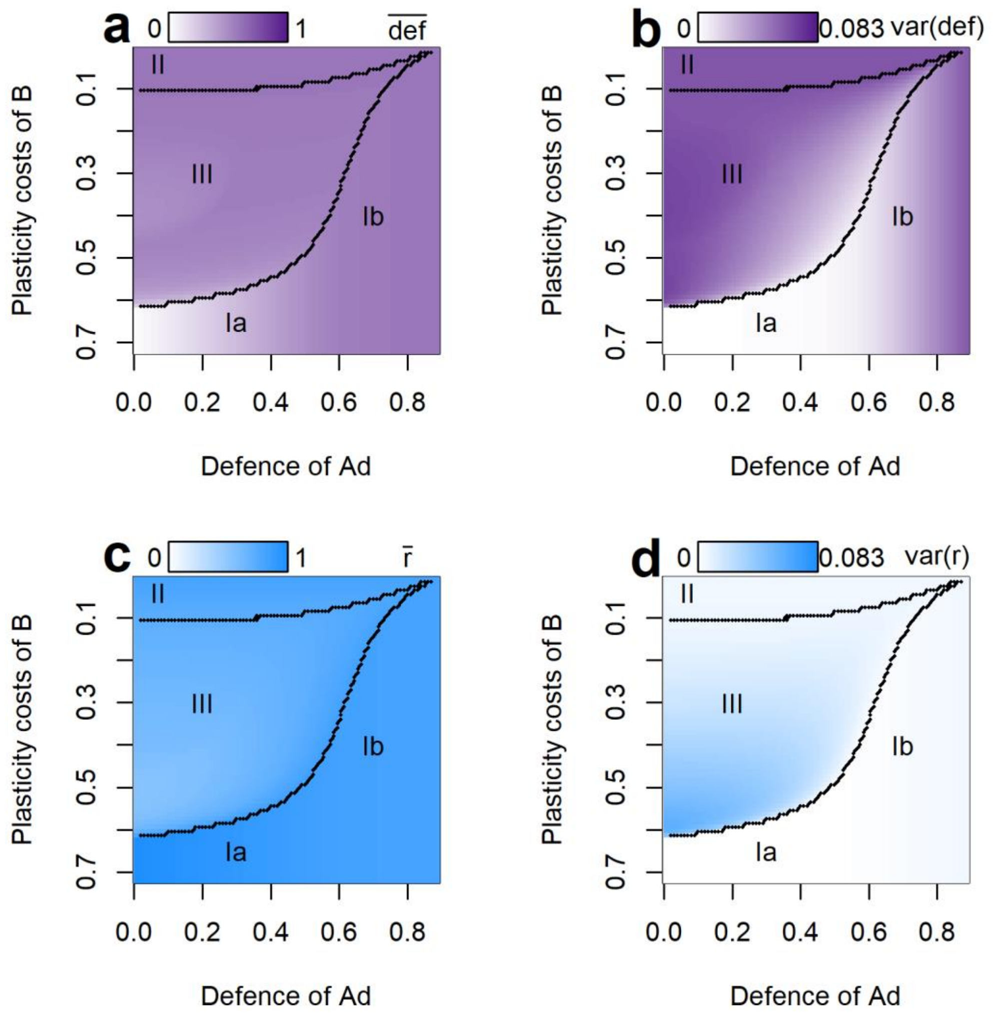

Figure S2: Trait values of the autotroph community *for scenario parallel 0.01*. Community mean defence (a), variance of the defence (b), community mean growth rate (c) and variance of the growth rate (d). Lines separate the regions I-III of different autotroph coexistence.

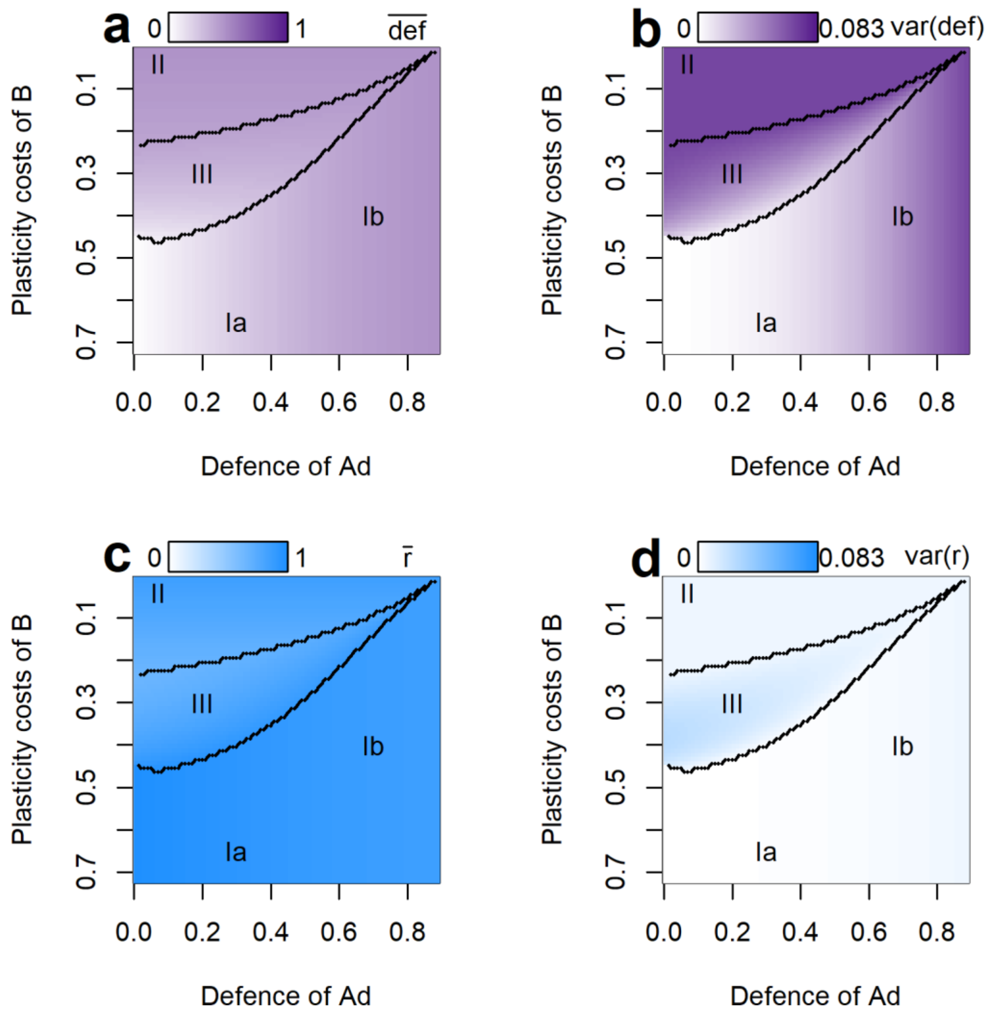

Figure S3: Trait values of the autotroph community for scenario *parallel 1*. Community mean defence (a), variance of the defence (b), community mean growth rate (c) and variance of the growth rate (d). Lines in separate the regions I-III of different autotroph coexistence.

Constellation *parallel*

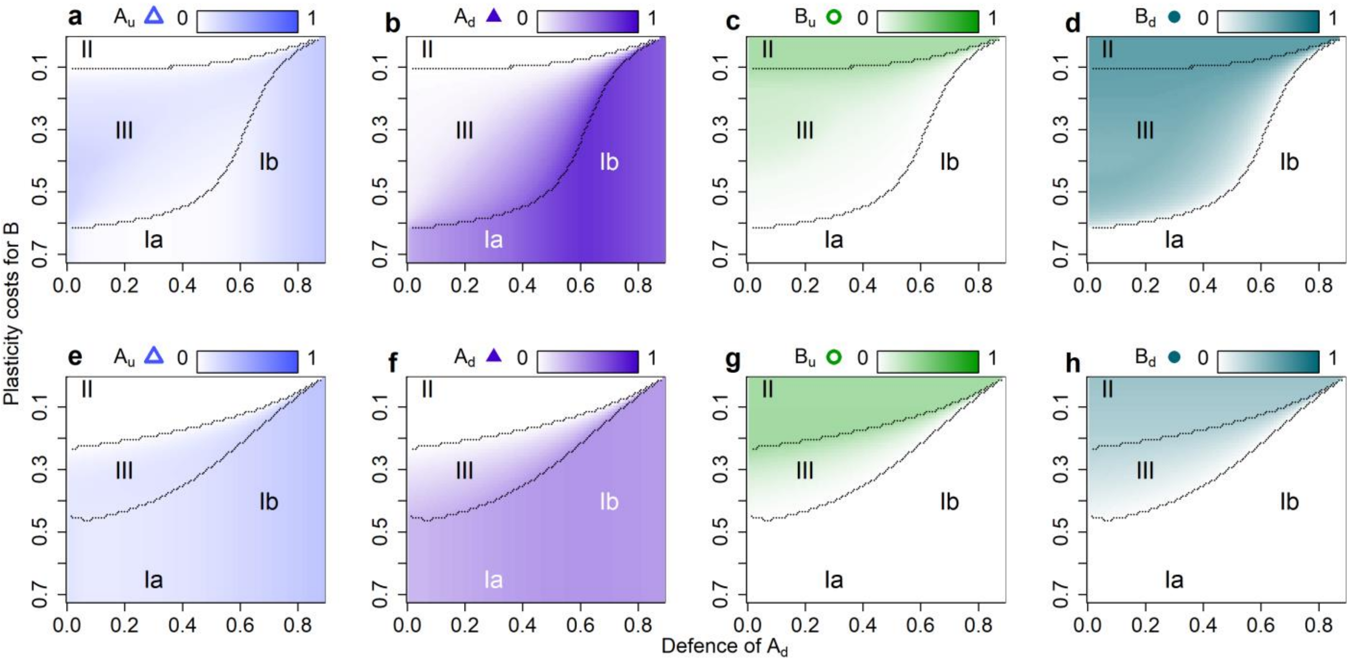

Figure S4: Biomasses for scenario parallel 0.01 and parallel 1. Biomasses of the four autotrophic phenotypes for scenarios parallel 0.01 (a-d) and parallel 1 (e-h) (higher biomasses are shown by darker colours). Lines separate the regions I-III of different autotroph coexistence.

Constellation *crossing*

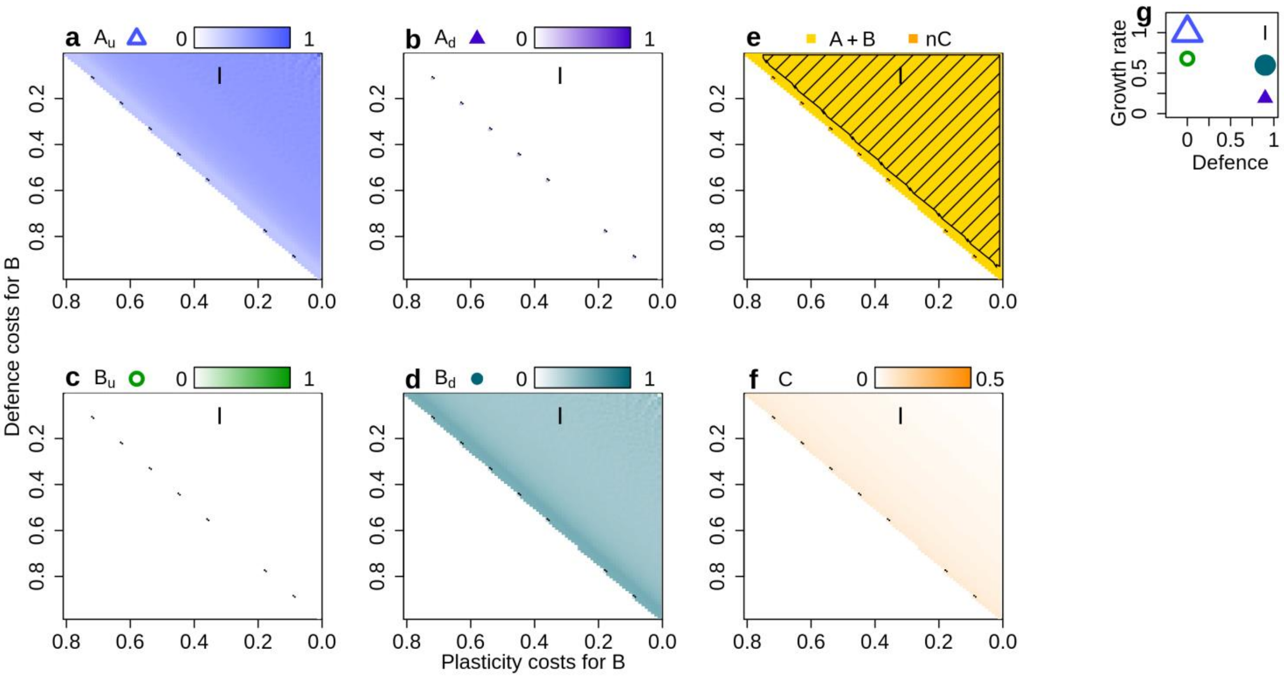

Figure S5: Biomasses and coexistence for the non-plastic scenario *crossing 0*. Biomasses of the four autotrophic phenotypes (a-d), their coexistence patterns (e), the consumer biomass (f) and an exemplary of the autotrophs' trait values (g) (higher biomasses are shown by darker colours). Larger symbols in g indicate the surviving phenotypes. Shaded areas in e depict oscillating systems (antiphase cycles in loose shading).

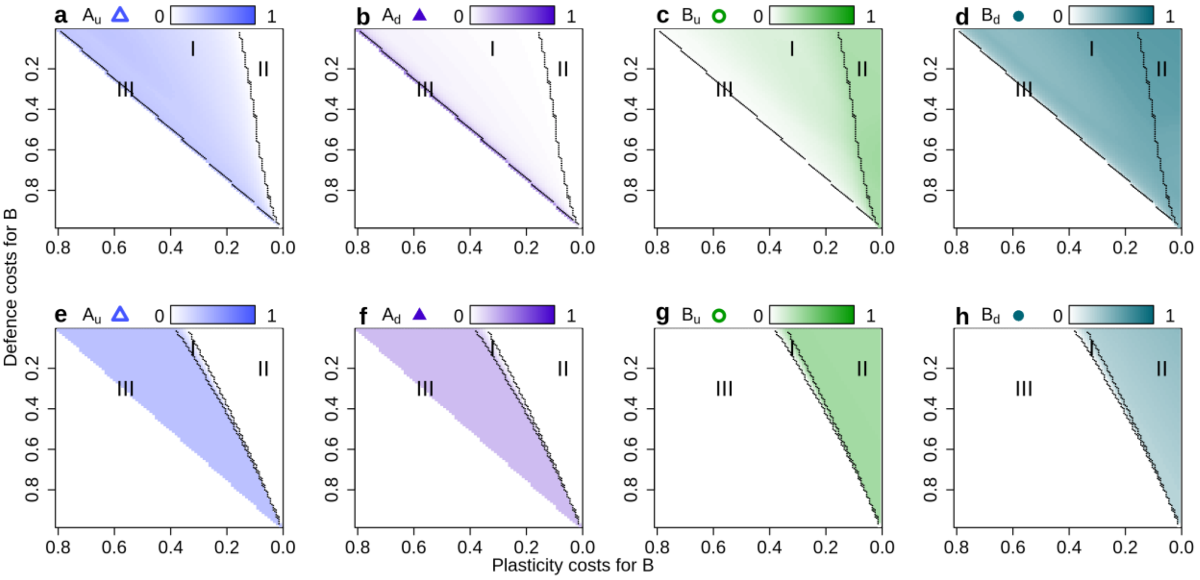

Figure S6: Biomasses for scenario *crossing 0.01* and *crossing 1*. Biomasses of the four autotrophic phenotypes for scenarios *crossing 0.01* (a-d) and *crossing 1* (e-h) (higher biomasses are shown by darker colours). Lines separate the regions I-III of different autotroph coexistence.

Constellation *angle*

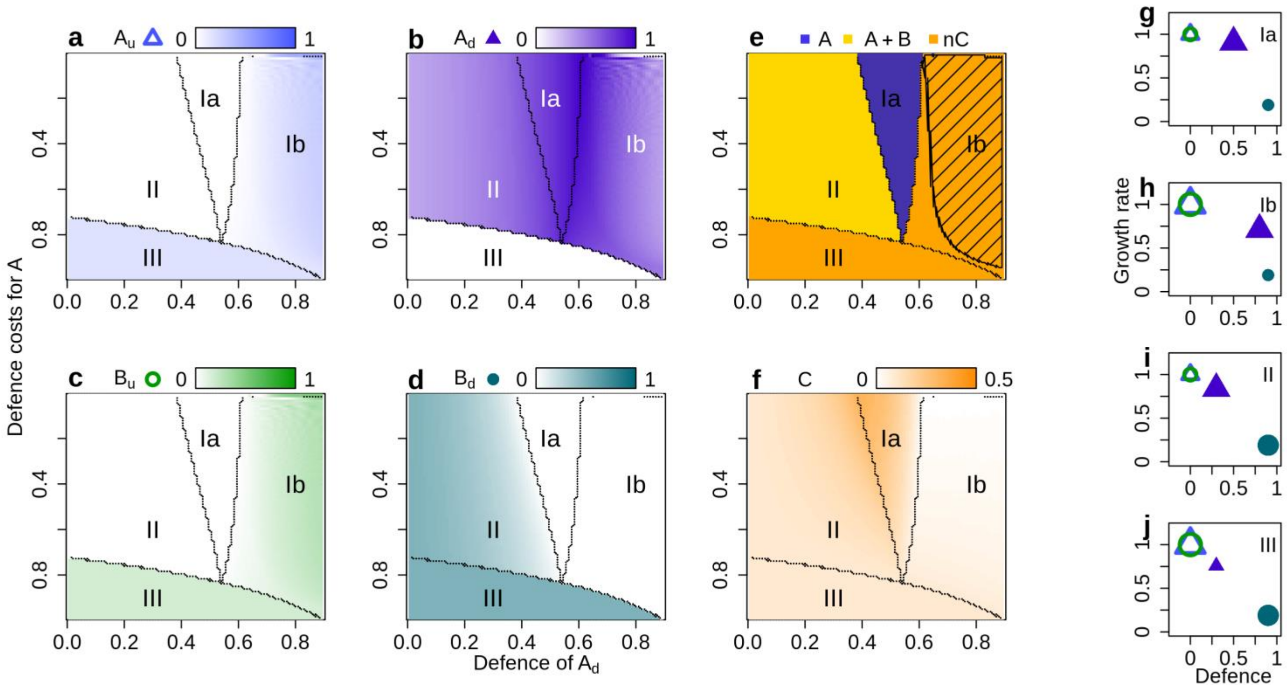

Figure S7: Biomasses and coexistence for the non-plastic scenario *angle 0*. Biomasses of the four autotrophic phenotypes (a-d), their coexistence patterns (e), the consumer biomass (f) and the autotrophs' trait values (g-j) (higher biomasses are shown by darker colours). Lines in a-f separate the regions I-III of different autotroph coexistence. An exemplary trait combination for every region is shown in g-j; larger symbols indicate the surviving phenotypes. Shaded areas in e depict antiphase cycles.

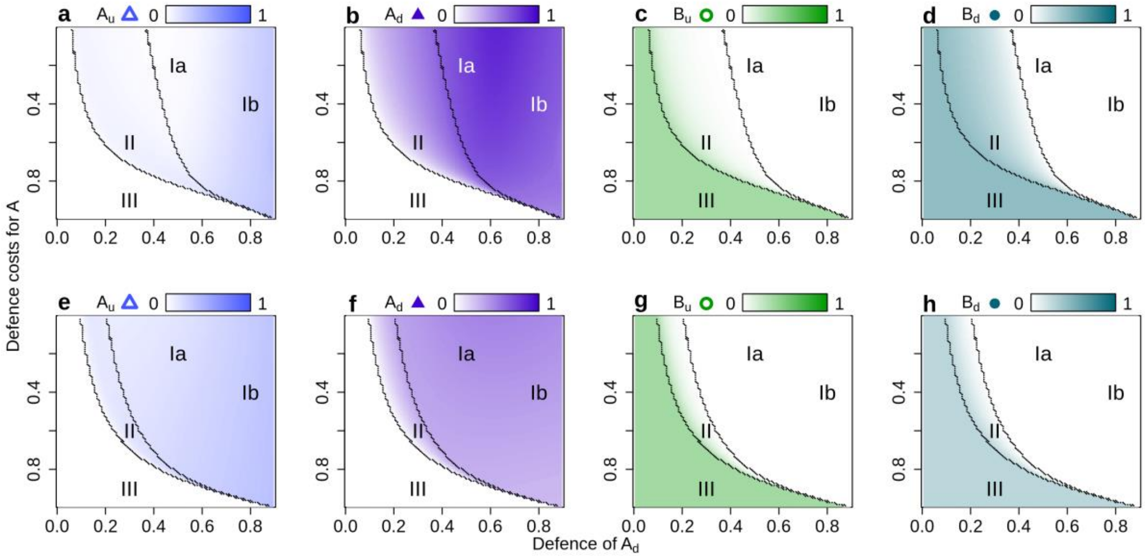

Figure S8: Biomasses for scenario *angle 0.01* and *angle 1*. Biomasses of the four autotrophic phenotypes for scenarios *angle 0.01* (a-d) and *angle 1* (e-h) (higher biomasses are shown by darker colours). Lines separate the regions I-III of different autotroph coexistence.

Impact of the shape of trade-off lines on coexistence

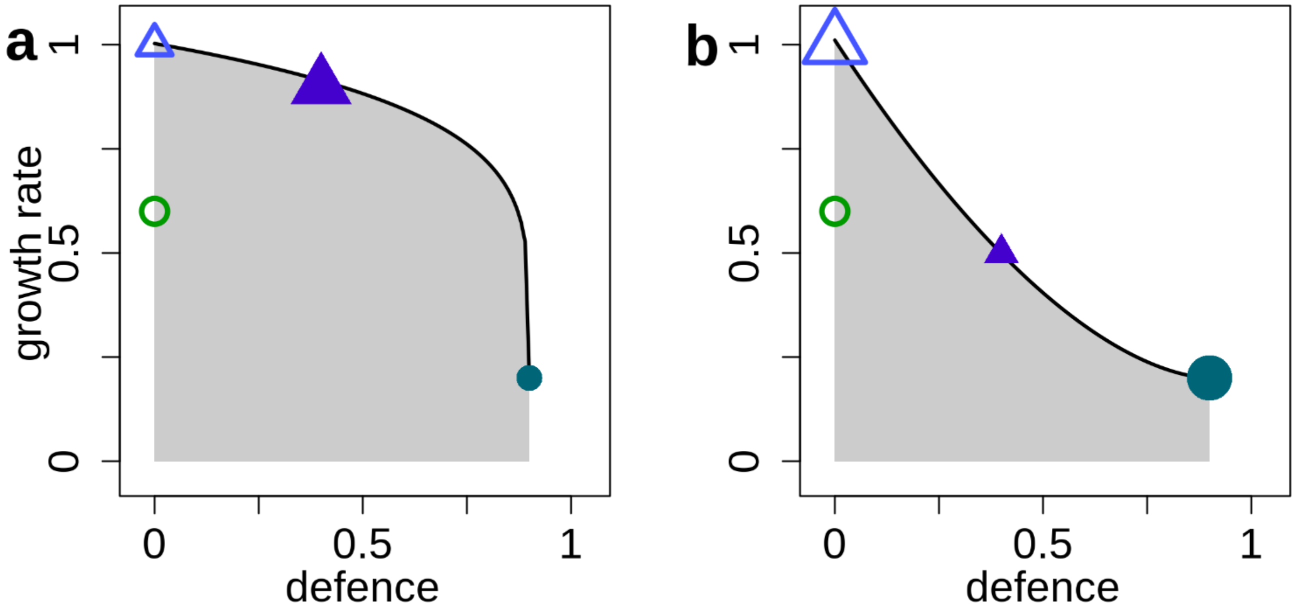

Figure S9: Concave (a) and convex (b) trade-off curves and the resulting surviving phenotypes. The trade-off curve (solid line) represents the boundary of the set of feasible trait combinations (gray area). The trait combinations with the highest fitness survive. If two or more trait combinations are of maximal fitness in the long term, the respective species with these trait combinations coexist (b), otherwise only one species survives (a). The shown trait combinations are examples, symbols denote different phenotypes. They are based on the scenario *parallel 0*, but with these parameters: defence of A_d = 0.4, defence costs dc_A = -0.25 for the concave and dc_A = -1.25 for the convex trade-off line, dc_B = -0.44, plasticity costs pc_B = 0.4.

Supplementary Information B: Derivation of maladaptive switching

Our results show that, in the long run, phenotypically plastic switching between phenotypes is nearly always maladaptive. In this appendix we show why this result must hold true if the system is at a stable equilibrium: if there is any switching at this equilibrium, this is always maladaptive, resulting in a source-sink dynamic between the two phenotypes. It is important to note that, in the initial (transient) phase of the dynamics where oscillations are still present, switching is typically adaptive (Fig. S10b, S10d). However, it always becomes maladaptive when an equilibrium has been reached (Fig. S10b, S10d). This means that, if there are ongoing oscillations, switching can in the long run still be adaptive (cf. Fig. 4C); but given the strongly stabilizing effect of inducible defences, we rarely found this as a long-term outcome in our models.

To explain why maladaptive switching inevitably arises at a stable equilibrium, we use here a simplified version of the model, with only a single autotroph A, which can express an undefended phenotype A_u and a defended phenotype A_d. In the two-autotroph food web we use in the main text, the mechanism underlying maladaptive switching is the same. We start by showing the equilibrium conditions in a model without switching, and then show how this is modified by phenotypic plasticity.

Single-autotroph model without switching

Without specifying the exact details of growth and consumption terms, a model with two autotroph phenotypes A_u and A_d and a single consumer C can be represented as follows:

$$\begin{aligned} \frac{dA_u}{dt} &= F_u \cdot A_u \\ \frac{dA_d}{dt} &= F_d \cdot A_d \\ \frac{dC}{dt} &= F_c \cdot C \end{aligned} \qquad \text{(S1)}$$

where F_u, F_d and F_c represent the fitness (i.e. the net per capita growth rate) of the undefended autotrophs A_u, defended autotrophs A_d, and consumers C, respectively. This system is at an equilibrium when all three equations are zero; if both autotroph phenotypes and the consumer all survive, this implies that $F_u = F_d = F_c = 0$.

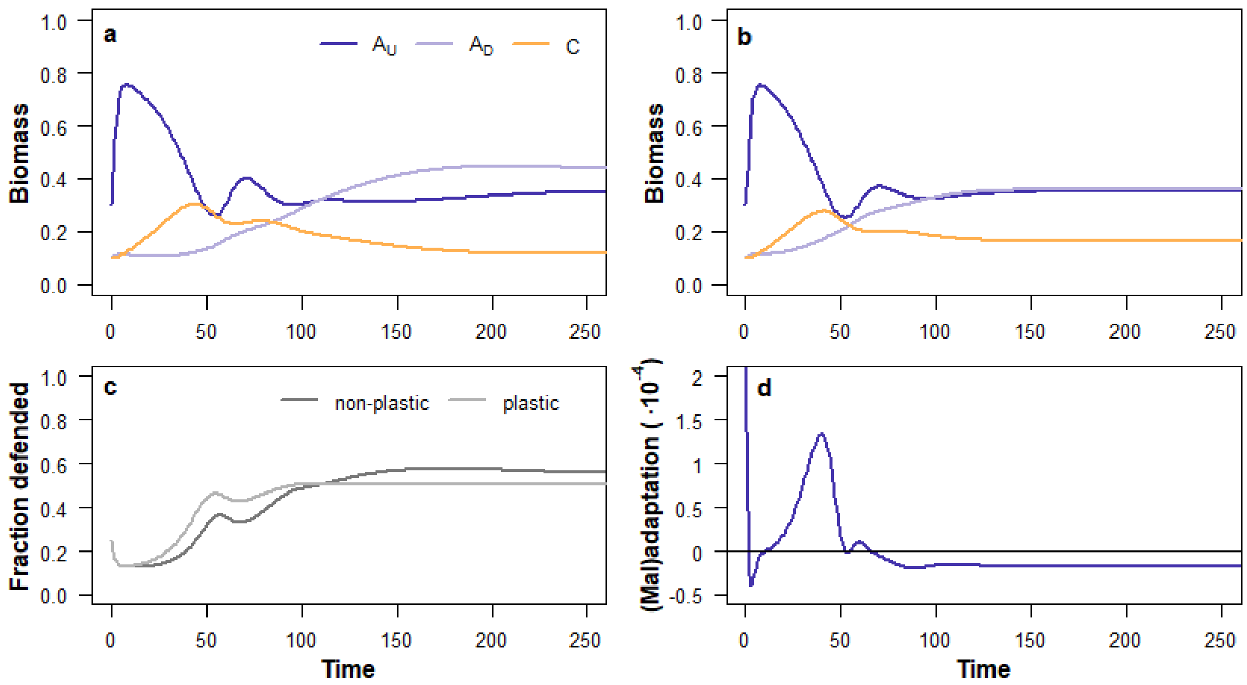

Figure S10: Example dynamics of the single-autotroph model without switching (a) and with switching (b). In both, dc_A = 0.85; in (b), χ_{max} = 0.01; all other parameters are identical to those for species A in the two-prey model (see Table S1). Comparing the dynamics of (a) and (b) shows the impact of switching on the location of the equilibrium: in (b), A_u is slightly higher and A_d is noticeably lower, together resulting in a higher consumer biomass. (c): Comparison of the fraction of defended phenotypes in the dynamics in (a-b): the plastic (switching) prey increases its level of defence more rapidly, resulting in a short-term benefit; however, its long-term level of defence is lower as a result of maladaptive switching towards undefended phenotypes. (d): The (mal-)adaptiveness index φ calculated from the dynamics in (b), showing that initially switching is adaptive, but becomes consistently maladaptive as the dynamics settle at their equilibrium state.

To understand the effect of switching in the next section, it is helpful to rewrite the first two equations to represent the change in total autotroph biomass ($A = A_u+A_d$) and the change in the frequency of the defended phenotype ($f = A_d/(A_u+A_d)$):

$$\begin{aligned} \frac{dA}{dt} &= \left(f \cdot F_d + (1-f) \cdot F_u\right) A \\ \frac{df}{dt} &= f \cdot (1-f) \cdot \left(F_d - F_u\right) \\ \frac{dC}{dt} &= F_c \cdot C \end{aligned} \qquad \text{(S2)}$$

In equation (S2), the meaning of F_u, F_d and F_c is the same as in the original equation (S1). f is the frequency of defended phenotypes in the autotroph population, and $(1 - f)$ is the frequency of undefended phenotypes. The second equation of S2 shows how fitness differences between the phenotypes will translate into changes in the frequency f: if $F_d > F_u$, defended phenotypes grow and reproduce faster than undefended ones, and the share of defended phenotypes will increase (e.g. between $t = 10$ and $t = 60$ in Fig. S10a). Conversely, if $F_u > F_d$, selection favours undefended phenotypes, which will then increase in frequency (e.g. between $t = 60$ and $t = 70$ in Fig. S10a).

The above equations are mathematically completely equivalent to those in equation (S1), and a stable equilibrium is again found when all equations are zero. From the second equation, we can see that coexistence of the two phenotypes (i.e. $f \neq 0$ and $f \neq 1$) implies that, at equilibrium, $F_u = F_d$ (i.e. the two phenotypes must have equal fitness); combined with the first equation, this implies that $F_u = F_d = 0$.

Single-autotroph model with switching

When we include the switching rates χ_u (switching from undefended to defended) and χ_d (from defended to undefended) in the model, the equations in (S2) now become:

$$\begin{aligned} \frac{dA}{dt} &= \left(f \cdot F_d + (1-f) \cdot F_u \right) A \\ \frac{df}{dt} &= \underbrace{f \cdot (1-f) \cdot (F_d - F_u)}_{selection} \underbrace{- f \cdot \chi_d + (1-f) \cdot \chi_u}_{switching} \end{aligned} \qquad \text{(S3)}$$

(The equation for the consumer C does not change, and is not included from this point.)

It can be seen that the switching rates do not directly affect the change in the total autotroph biomass A, since undefended and defended individuals are counted there as a single population. Instead, they affect the frequencies: χ_u increases the frequency of defended phenotypes f (since it represents the switching from undefended to defended phenotypes), while χ_d decreases f (since it represents switching from defended to undefended phenotypes). The inclusion of these additional terms has the effect of enabling a faster shift in phenotypes when consumer biomass changes (see Fig. S10c, $t < 50$: the increase in C induces a much more pronounced shift towards defended phenotypes in the plastic prey). Phenotypic plasticity is thus clearly advantageous under temporally variable conditions, as it is also indicated by the strongly positive value for φ in this part of the dynamics (Fig. S10d). However, this is not the case when the dynamics converge towards an equilibrium. In the above equations (S3), just as before, the system

is at a stable equilibrium when all equations are zero. Without switching, this always means that $F_u = F_d = 0$ (see previous section); but here, this is modified by the switching rates to:

$$\underbrace{f \cdot (1-f) \cdot (F_d - F_u)}_{selection} = \underbrace{f \cdot \chi_d - (1-f) \cdot \chi_u}_{switching} \quad \text{(S4)}$$

In more intuitive terms: the frequency of defended phenotypes (second line in equation (S3)) does not change when the effects of selection and of switching balance each other out. While it is in principle possible for this to happen because both terms in eq. (S4) are zero – i.e. the phenotypes have equal fitness, and there is no net switching between them – we never observed this in our simulations. Instead, in the model with switching, the populations converged to a slightly different equilibrium (compare Fig. S10b with S10a). At this equilibrium, the phenotypes do not have equal fitness: in the example shown in Fig. S10b, from $t \approx 150$ onwards, consumer biomass is higher than it would have been without switching (Fig. S10a), indicating that $F_d > F_u$. Thus, both terms in eq. (S4) are positive: at the equilibrium, selection continuously "pushes" towards a higher share of defended phenotypes, since these have a higher fitness; but this is counteracted by net switching from defended to undefended phenotypes. Since selection is, by definition, always adaptive (i.e. it results in an increase in the frequency of whichever phenotype has a higher fitness) this means that the net effect of switching at equilibrium must be maladaptive.

The degree of maladaptive switching at equilibrium may be weak compared to the positive short-term effects seen in the initial phase (see Fig. S10d). But since the positive (adaptive) effects of switching are only transient, it is the maladaptive effect that plays out in the long-term, and thus affects properties such as coexistence or consumer biomass. Of course, the above argument does not apply when there are ongoing oscillations, and in this case switching can on average still be adaptive in the long run (cf. Fig. 4C), but this happened only in a very small minority of our simulations.
